# Structural and evolutionary constraints shape adaptive landscapes of immune-related genes across mammalian phylogeny

**DOI:** 10.1371/journal.pone.0332734

**Published:** 2025-11-07

**Authors:** Zhengtian Li, Mubbashar Hassan, Hafiz Ishfaq Ahmad, Muhammad Adnan Ashraf, Akhtar Rasool Asif, Iram Qadeer, Abid Hussain Shahzad, Shaista Abbas, Muhammad Sajid, Abdul Mateen, Irfan Ahmed, Jamal Muhammad, Sayyed Aun Muhammad, Farid S. Ataya

**Affiliations:** 1 College of Biological Resource and Food Engineering, Qujing Normal University, Yunnan, 655011, China; 2 Department of Clinical Sciences, Theriogenology Section, College of Veterinary and Animal Sciences, Jhang, Sub Campus UVAS, Lahore, Pakistan; 3 Department of Animal Breeding and Genetics, Faculty of Veterinary and Animal Sciences, The Islamia University of Bahawalpur, Pakistan; 4 Institute of Microbiology, Faculty of Veterinary Science, University of Veterinary and Animal Sciences, Lahore, Pakistan; 5 Department of Animal Sciences, College of Veterinary and Animal Sciences, Jhang, Sub Campus UVAS, Lahore, Pakistan; 6 College of Animal Sciences and Technology, Huazhong Agricultural University, Wuhan, China; 7 Department of Zoology, The Govt. Sadiq College Women University, Bahawalpur, Pakistan; 8 Department of Basics Sciences, Physiology Section, College of Veterinary and Animal Sciences, Jhang, Sub Campus UVAS, Lahore, Pakistan; 9 Department of Pathobiology, College of Veterinary and Animal Sciences, Jhang, Sub Campus UVAS, Lahore, Pakistan; 10 Department of Clinical Sciences, College of Veterinary and Animal Sciences, Jhang, Sub Campus UVAS, Lahore, Pakistan; 11 Department of Animal Nutrition, Faculty of Veterinary and Animal Sciences, The Islamia University of Bahawalpur, Pakistan; 12 Department of Parasitology, Faculty of Veterinary Sciences, Cholistan University of Veterinary and Animal Sciences, Bahawalpur, Pakistan; 13 Department of Biochemistry, College of Science, King Saud University, PO Box 2455, Riyadh, 11451, Saudi Arabia; Shantou University Medical College, CHINA

## Abstract

The evolutionary dynamics of immune-related genes GBP5, GZMB, IFNG, IRF7, KLRD1, RTP4, TNFSF4, and TRAT1 were investigated through comprehensive phylogenetic and selection analyses across mammalian species. Using concatenated gene sequences, we applied advanced methods including PAML (a software tool that analyzes evolutionary selection pressures by comparing rates of genetic changes), MEME (a method to identify patterns in protein sequences that may indicate functional sites), and structural modeling (a technique to predict 3D protein shapes) to assess co-evolution and adaptation. Site- and branch-specific selection tests revealed widespread positive selection (ω > 1), with 15–26 branches showing statistically significant adaptive evolution (p < 0.05), particularly in functional domains critical for pathogen recognition and immune regulation. Recombination analysis identified gene-specific patterns, with GBP5, GZMB, and IRF7 exhibiting significant recombination breakpoints, while IFNG and TNFSF4 remained conserved. Functional annotation highlighted the biological relevance of selected sites, linking them to inflammasome activation (GBP5), apoptotic pathways (GZMB), interferon signaling (IFNG, IRF7), and lymphocyte regulation (KLRD1, TNFSF4). Tissue-specific expression analysis confirmed these genes’ roles in immune-active tissues, with enriched pathways including Th1/Th2 differentiation (KEGG hsa04658) and cytokine regulation. These findings underscore the persistent evolutionary arms race between hosts and pathogens, with immune genes adapting to maintain effective defense mechanisms. The study provides a framework for understanding mammalian immune gene evolution, offering insights into conserved functional domains that may inform therapeutic targeting and vaccine design. By integrating phylogenetics, selection analysis, and functional genomics, we elucidate the molecular signatures of adaptation in key immune regulators, advancing our knowledge of host-pathogen coevolution.

## Introduction

The gastrointestinal tract of mammals is a dynamic ecosystem with a high population of billions of bacteria known as the gut microbiota. Animal gut microbiota is a complex symbiotic ecosystem that undergoes continuous fluctuations [1]. Environmental factors, including energy sources and changes in the niche induced by microbial colonizers, influence dynamic changes [2]. Microorganism growth is supported by carbon sources in the host’s food and shedding epithelial cells. The quantity of viable microbial biomass is restricted by intestinal secretions and peristalsis [3]. Understanding the boundaries of microbiota stability is essential for appropriately modeling biological systems and diseases in live organisms [4]. Inbreeding can create animals with identical genes in the host. Still, the microbiota, the microorganism community in the host, varies based on factors like the supplier, housing facility, and specific cages used for experiments. Differences in the microbiota in various colonies of inbred or targeted mouse models can explain the differences in observed phenotypic outcomes across different research facilities [5]. In other cases, the dominance of potent traits from one hazardous microbe could surpass any differences in the microbiota’s makeup. Yet, in other cases, variations in strains within a single organism might affect the interaction between the host and microbes in a mutually advantageous manner [6]. Establishing a genetically homogeneous colony of mice can be achieved by standardizing the microbiota in their intestines, which has been comprehensively sequenced and encompasses a wide variety of microbial species [7]. This aimed to provide uniform and reproducible research projects across different periods and research institutes. Existing studies on the microbiota have mostly been carried out for brief durations and have predominantly concentrated on certain species [8–10]. Numerous seminal studies have established the importance of gut microbiota for the growth and operation of the adaptive immune system. Segmented filamentous bacteria (SFB) in the gut play a key role in the differentiation of Th17 cells, a subset of T helper cells implicated in autoimmune disease development and pathogen defense [11]. This study demonstrated that Th17 cells were significantly lower in germ-free mice, who do not have a normal microbiota, highlighting the importance of gut bacteria in fostering the development of particular immune responses. Round and Mazmanian (2010) further demonstrated that the commensal bacterium Bacteroides fragilis produces polysaccharide A (PSA), which is essential for controlling the ratio of pro-inflammatory Th17 cells to anti-inflammatory regulatory T cells (Tregs). This study highlighted the importance of the microbiota in preserving immunological homeostasis and offered insights into how particular microbial compounds can alter the immune response [12]. Despite the fact that most studies highlight the advantageous function of gut bacteria in fostering adaptive immunity, there is some contradicting data. For example, Brown et al. (2019) questioned the universality of gut microbiota-induced Th17 cell activation, speculating that host factors or other microbial communities may have varying effects on this process. It suggests that the interaction between microbiota and adaptive immunity may be more context-dependent than previously believed. Their study found that in certain germ-free mice colonized with human microbiota, there was no rise in Th17 cells [13].

The humoral immune response, comprising antibodies, cytokines, and other soluble proteins, is an essential component of the host immune system that interacts with the gut flora. It is crucial in protecting against infections [14]. The reciprocal relationship between the host and the gut microbiota has garnered increasing attention in the scientific community as a co-evolutionary link. Internal and external evolutionary pressures over millions of years have shaped the genetic compositions of host and microbial communities [15]. This has resulted in a delicate equilibrium that improves the overall health and flexibility of the host organism [16]. A thorough understanding of how the molecular evolution of the gut microbiota intersects with the selection pressures impacting the host immune system, particularly concerning humoral immunity, is lacking despite distinct investigations on these topics. The guanylate-binding protein family, which includes GBP5 (Guanylate Binding Protein 5), is involved in some physiological functions, including the immunological response.

The guanylate-binding protein family, which includes GBP5 (Guanylate Binding Protein 5), is involved in some physiological functions, including the immunological response. GBP5 controls inflammasome activation, a critical mechanism for cleaving and activating inflammatory cytokines like IL-1β, in response to bacterial and viral pathogens [17]. The main sources of the protease GZMB (Granzyme B) are natural killer cells and cytotoxic T lymphocytes. GZMB is a key effector molecule that induces apoptosis in virus-infected cells and tumor cells by cleaving and activating executioner caspases [18]. IFNG (Interferon Gamma) is a cytokine that plays a central role in innate and adaptive immune responses. It is a potent activator of macrophages, driving antimicrobial activity and antigen presentation primarily through the JAK-STAT signaling pathway [19]. A transcription factor called IRF7 (Interferon Regulatory Factor 7) controls how type I interferons are expressed in response to viral infections. IRF7 is the master regulator of the type I interferon (IFN-α/β) response, amplifying interferon production upon viral detection [20]. Natural killer cells and certain T cell subsets carry a protein encoded by KLRD1 (Killer Cell Lectin-Like Receptor Subfamily D, Member 1). KLRD1 (CD94) forms complexes with NKG2 family members to recognize HLA-E molecules, playing a vital role in regulating NK cell cytotoxicity and cytokine production [21]. Immune signaling pathways involving RTP4 (Receptor Transporter Protein 4) may impact how the body reacts to gut microorganisms or microbial antigens. TNFSF4 (Tumor Necrosis Factor Superfamily Member 4) is a co-stimulatory molecule expressed on activated antigen-presenting cells. It binds to the OX40 receptor on T cells, providing a critical secondary signal that promotes T cell survival, effector function, and the development of memory [22]. TRAT1 (T Cell Receptor-Associated Transmembrane Adaptor 1) is involved in T cell receptor signaling and development. It plays crucial roles role in gut microbe interactions T cells in maintaining gut immune homeostasis and regulating responses to gut microbes [23].

Humoral immunity is a crucial element of the adaptive immune system. It produces antibodies and orchestrates immunological reactions against various illnesses [24]. The immune system faces continuous challenges, leading to an ongoing interaction between the host and its gut flora. The host’s immune system influences the microbial populations in the gastrointestinal tract through selection forces. The gut microbiota influences the host’s immune system through many mechanisms that regulate immunological equilibrium and tolerance [25,26]. Advancements in high-throughput sequencing technology and bioinformatics tools have greatly enhanced our ability to analyze the intricate molecular processes involved in host-microbiota interactions. Utilizing these approaches provides extraordinary opportunities to examine the genetic traits of both hosts and the microorganisms within them [27,28]. This enables a more in-depth research of the mutually important dynamics that have altered the ecology of the mammalian gut. These methods allow for a thorough examination of how the host’s humoral immune system affects the gut microbiota’s genetic development [29]. This study investigates several fundamental problems related to the co-evolution of the host and the microbiota. What molecular changes do gut microbiota undergo in response to the host’s humoral immune responses? How do these adaptations differ among various mammalian species? Do preservative mechanisms or distinctive characteristics exist that establish the co-evolutionary connection between the host’s humoral immunity and the gut microbiota? To investigate these inquiries, we will extensively examine the genetic variation present in the gut microbiota of several mammalian species. We aim to use advanced bioinformatics approaches to find genetic patterns that show positive selection. This will help us understand how the host’s immune system affects the gut microbiota in terms of evolution. This research aimed to investigate the rapid evolution of GBP5, GZMB, IFNG, IRF7, KLRD1, RTP4, TNFSF4, and TRAT1 genes and explain the significant sequence divergence found in different animal species. We showed that these genes underwent rapid evolution due to positive selection. We examined that genes evolved rapidly due to positive selection. These genes influence the balance between gut homeostasis and immunological-mediated disease, which play various roles in the immune response to gut bacteria, including immune cell activation, cytokine synthesis, and immune control. It is essential to comprehend their roles in gut microbiota to clarify the mechanisms behind host-microbe interactions and their effects on health and disease,

## Materials and methods

### Data collection

The sequences of the coding sections of the GBP5, GZMB, IFNG, IRF7, KLRD1, RTP4, TNFSF4, and TRAT1 genes utilized in the analysis were obtained from NCBI. To capture a broad spectrum of mammalian evolutionary history, we analyzed a total of 42 species representing 12 different orders (S1 Table). This approach aimed to reduce phylogenetic bias and provide a more generalizable understanding of the evolutionary processes under investigation. The included orders represent major clades such as Euarchontoglires (Primates, Rodentia, Lagomorpha, Scandentia, Dermoptera), Laurasiatheria (Cetartiodactyla, Carnivora, Chiroptera, Perissodactyla, Eulipotyphla), and Afrotheria, as well as representatives of marsupials (Didelphimorphia). We used gene sequences from the genomes of representatives of various mammalian species. The whole-genome sequences were retrieved from the Ensembl database. The amino acid and nucleotide sequences of GBP5, GZMB, IFNG, IRF7, KLRD1, RTP4, TNFSF4, and TRAT1, which are important for gut microbiota adaptation, are expressed in mammalian species. Ensembl was accessed to get the coding sequences for these genes, all covering various mammalian species. This was done based on the gene annotation (two conserved neighboring genes) [30]. The BLASTn v2.2.29 + algorithm chose the most effective scaffold [31]. Checking for a start and stop codon was part of the annotation process that was carried out with MITOS [32]. MACSE v1.01b [33] and ClustalW v2 aligned the protein-coding and ribosomal genes [34]. We eliminated any genes that were less than a third of the length of the overall locus alignment. To capture a broad spectrum of mammalian evolutionary history, we prioritized species representing different branches of the mammalian phylogenetic tree. This approach aimed to reduce phylogenetic bias and provide a more generalizable understanding of the evolutionary processes under investigation. We included species from various orders, such as Primates, Carnivora, Rodentia, and Artiodactyla, ensuring various ecological and physiological adaptations.

### Interspecific sequences alignments

The nucleotide sequences of the GBP5, GZMB, IFNG, IRF7, KLRD1, RTP4, TNFSF4, and TRAT1 genes were aligned separately using the ClustalW tool within the MEGA software v.7.0.14 [35] using the default parameters. The Gblocks v.0.91b software [36] was employed with standard settings to eliminate inaccurately aligned regions and differing segments.

### Phylogenetic analysis

PartitionFinder v.1.1.1 [37] was utilized to determine each partition’s optimal partitioning scheme and substitution models before conducting phylogenetic analysis. This was based on the Akaike (AIC), corrected Akaike (AICc), and Bayesian (BIC) information criteria. The GTR + 0 + I model is the most suitable for molecular evolution [38]. RAxML (version 8.2.12) [39] generated the maximum probability unrooted tree with 10,000 bootstrap replicates. It was unable to designate an outgroup for tree construction since orthologs of the GBP5, GZMB, IFNG, IRF7, KLRD1, RTP4, TNFSF4, and TRAT1 genes were not found in the genomes of other organisms. Phylogenetic trees were constructed using gene sequence data from the mammalian GBP5, GZMB, IFNG, IRF7, KLRD1, RTP4, TNFSF4, and TRAT1 genes to show the evolutionary connections and alterations in these genes over time. Phylogenetic trees were created using MEGA (Molecular Evolutionary Genetics Analysis) version 10.0.5 [35] using a maximum likelihood method. The topology of the tree we built with the neighbour-joining method was evaluated by applying the maximum likelihood method to the Whelan and Goldman (WAG) substitution model [40]. To further evaluate the stability of the tree structure, we conducted 1000 bootstrap repetitions. Gene trees and other phylogenetic trees can be compared and evaluated precisely using the TreeBeST-generated species tree as a benchmark (http://treesoft.svn.sourceforge.net/viewrc/treesoft/) [41].

### Recombination analysis

Recombination analysis was performed using the GARD (Genetic Algorithm for Recombination Detection) method [42] to identify potential recombination breakpoints that could confound selection analyses. The AICc score was used for model selection. A finding of significant recombination was supported if the multi-tree model (allowing different topologies between segments) was favor\ed over the single-tree model by an evidence ratio of 100:1 or greater (ΔAICc ≥ 10), indicating genuine topological incongruence rather than mere rate variation [43]. Using likelihood ratio tests (LRTs), opposing models were compared to identify the best-fitting model among M8 vs. M8a and M2 vs. M2a. The degrees of freedom (df) were calculated by subtracting the free parameters in the compared models [44]. Positive selection was shown by identifying codons with more than one dN/dS ratio (ω).

### Codon-based positive selection analyses

We used site models (M1, M2, M8a, and M8) that allowed variation between sites to determine the chance of each site in each gene being under positive selection [45]. This was done to assess the probability of occurrence for each location inside each gene. This model detected signs of positive selection within the gene at a few specific locations during brief periods of evolutionary time. Both the alternative model of positive selection (*ω > 1*) and the null model of neutral evolution (*ω = 1*) were employed in the branch-site test to assess selective pressure on each branch. The alternative model of positive selection was selected due to its prediction that each branch will experience greater degrees of selection compared to the null model. We used this methodology to identify examples of positive selection on several genomic sites across all grasshopper lineages. We employed the likelihood ratio test (LRT) to assess each paired model and chose the one that most closely fits our data.

The optimal value of the Codon parameter was determined in the M1 model, with Hominidae as a foreground clade, based on AIC, AICc, and BIC criteria. The parameter specifies the equilibrium codon frequencies in the codon substitution model. The correct branch lengths of the phylogenetic tree for the codon-based analysis of positive selection were calculated using model M0 with fixed length = 0. The branch lengths were then fixed for all experiments with fix length = 2. Branch-site tests were conducted on 35 specific branches and clades of the mammalian phylogenetic tree using strict (χ 2-distribution of LRT statistics, P < 0.01) and relaxed conditions (50:50 mixture distribution of the χ 2-distribution and a point mass of zero, P < 0.05). The lenient settings were used to reduce the chance of a false-negative error, as the test is cautious under strict conditions [46]. The Bonferroni correction and the Benjamini-Hochberg procedure were used to minimize the chances of a false-positive error caused by multiple tests [47]. The BEB technique was utilized over a significant period of the LRT to identify codons likely to undergo positive selection, with PP criteria set at 0.9 and 0.95 [48]. The IBS program version 1.0 showed the localization of areas experiencing positive selection pressure in protein primary structures [49]. We conducted further positive selection tests using the MEME program within the HyPhy software package v.2.2.4 [50] to confirm the reliability of our results.

We compared the ratio of synonymous to non-synonymous substitutions (dN/dS) to determine whether selective pressure acted on homologous nutritional pathway genes. The ω was calculated using the PAML (version 4.9j) codon-based ML approach called CODEML [51]. We used two different PAML models to determine whether there was a difference in the selective pressures exerted on the various grasshopper lineages. In this analysis, we focused on the ω values at the ends of the branches. We focused on the rate at which mutations have accumulated between modern species and their closest reconstructed relatives. According to the free-ratio model [52], the & values at each branch are predictably random. Initially, positive selection was detected using the branch-site model in PAML [53]. The parameters for testing the null hypothesis were *ω = 1*. The level of statistical significance was determined by employing a chi-square distribution, with the difference in the number of parameters for the two models being equal to two times the difference in the log-likelihood values and the degrees of freedom. The identification of positive selection is frequently inconsistent due to different approaches regarding periods, assumptions, methodologies, and gene conversion bias [54]. The PAML site-branch model has been adjusted for multiple tests using Bonferroni’s correction with various parameters.

Furthermore, we validated these findings using various independent tools, including the HyPhy package (version 2.5.31) [55]. We used site models (M1, M2, M8a, and M8) that allowed variation between sites to determine the chance of each site in each gene being under positive selection. This was done to assess the probability of each position within each gene. This model detected signs of positive selection within the gene at a few specific locations during brief periods of evolutionary time [56]. Both the alternative model of positive selection (ω > 1) and the null model of neutral evolution (ω = 1) were employed in the branch-site test to assess if each branch experienced selective pressure. The alternative model of positive selection was selected for its prediction of higher selection levels on each branch compared to the null model. We used this methodology to identify examples of positive selection on several genomic sites across all grasshopper lineages. We employed the likelihood ratio test (LRT) to assess each paired model and chose the one that most closely fits our data.

Statistical tests were conducted using the CODEML algorithm in the PAML software package v.4.8 [42] to assess adaptive evolution... Site models (M8, M8a) and branch-site models (Test 1: Model A vs. A; Test 2: Model A1 vs. A) were employed [43]. Likelihood ratio tests (LRTs) were used to compare these nested models. The significance of the LRT statistic was assessed using a χ² distribution with the appropriate degrees of freedom. To account for multiple testing, we applied both the Bonferroni correction and the Benjamini-Hochberg FDR procedure [46]. For branch-site tests, we used both a strict significance threshold (p < 0.01) and a more lenient threshold (p < 0.05) to reduce the chance of false negatives [45]. Positively selected sites were identified using the Bayes Empirical Bayes (BEB) method [47], with posterior probabilities PP > 0.90 considered significant. To confirm the reliability of our PAML results, we conducted additional tests using the aBSREL method within the HyPhy software package v.2.2.4 [49], which tests for episodic diversifying selection on a per-branch basis. For aBSREL, significance was assessed at p < 0.05 after correction for multiple comparisons (FDR).

### Protein domain and structure analysis

The positively selected sites from the previous stage were used for future structural analysis. We utilized the protein secondary structure prediction program PSIPRED 4.0 (version 4.0) [57] and the AlphaFold2 protein structure database [58] to generate educated guesses about the degree of similarity between the mammalian proteins’ predicted secondary and tertiary structures. SCANSITE 4.0 was used to develop predictions for the specific sites of kinase phosphorylation and binding domains, given a database of 81 mammalian kinases/domains [59]. The output was then filtered through an additional phase with the rigor level set to “high.” After that, the linker sections and the domains were examined by hand. To further understand the functional significance of the putatively selected locations, we superimposed them on the 3D structures of the proteins. Using the homology modeling software made available by the MODELLER (version 9.23) and I-TASSER server (version 5.1), we made predictions about the 3D gene structures [60]. The mammalian genome, received from GenBank, was used to deduce the protein sequences of positively chosen genes. From UniProt [61], we collected functional information regarding the presumptively recognized genes as being positively selected.

### Functional analysis

The protein sequences were evaluated using two free tools found online: Clustal W was used for sequence alignment, and the LPIcom server was used to annotate amino acid similarities. This protein was analyzed with the help of the online LPIcom server [62]. We classified the detected proteins based on their projection at a particular gene ontology (GO) hierarchy level, emphasizing the GO ‘Biological Process’ (GOBP) class. To do this, we used the function ‘groupGO,’ which you can find here. The ‘enrichGO’ function was then used to execute enrichment tests for GOBP keywords based on a protein kinase distribution against a background list of all proteins in the relevant annotation database. These tests were conducted against a protein kinase distribution. To visualize all GO terms related to nutrient metabolism, we used g: GOSt, a web tool in the g: Profiler suite (http://biit.cs.ut.ee/gprofiler/), [63] in conjunction with Cytoscape’s Enrichment Map program (http://www.baderlab.org/Software/EnrichmentMap) [64]. We integrated information from these large-scale transcriptome investigations with that from the Genotype-Tissue Expression (GTEx) database Release V8 (dbGaP Accession phs000424.v8.p2) [65]. This database offers information on gene-level associations that explain how gene expression levels test and mediate impacts on phenotypes [66].

## Results

**Fig 1:**
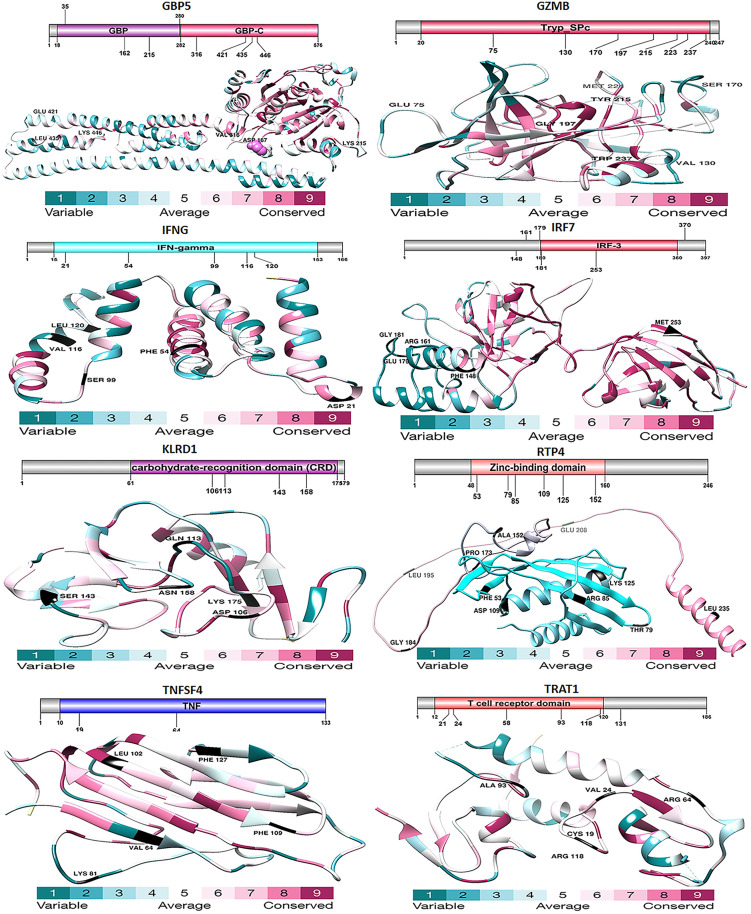
Analysis of the domain structure and selection of the proteins Trat1, Gbp5, Ifng, Irf7, Klrd1, Rtp4, and Tnfsf4. This diagram, generated using the DOG 1.0 illustrator, displays the structural organization of the GBP5, GZMB, IFNG, IRF7, KLRD1, RTP4, TNFSF4, and TRAT1 proteins, with a focus on the examination of their conserved domains. Emphasis is given to the protein domains, specifically identifying regions subject to positive selection. These sites are correlated with the three-dimensional configuration of proteins, exposing the adaptive evolution occurring at the molecular scale.

We employed the MirrorTree method (Ochoa and Pazos, 2010) to confirm the co-evolution of the GBP5, GZMB, IFNG, IRF7, KLRD1, RTP4, TNFSF4, and TRAT1 genes. We computed the Pearson correlation coefficients for the evolutionary distance matrices of phylogenetic trees obtained from multiple sequence alignments of orthologous genes from several mammalian species. The Pearson correlation coefficient varied between 0.84 and 0.95, with a significance level 0.001. Phylogenetic trees were compared in pairs, showing high Pearson correlation coefficients, which confirmed the co-evolution of the GBP5, GZMB, IFNG, IRF7, KLRD1, RTP4, TNFSF4, and TRAT1 genes. We utilized concatenated gene sequences that were matched to create a phylogenetic tree. Employing concatenated gene sequences instead of individual gene sequences increased the statistical power of the molecular evolution study. It improved the accuracy of the resulting phylogenetic tree by analyzing a more significant number of substitutions. We generated an unrooted phylogenetic tree by merging the coding sections of the GBP5, GZMB, IFNG, IRF7, KLRD1, RTP4, TNFSF4, and TRAT1 genes (Fig 1). The terminal nodes of the phylogenetic tree were strongly supported by bootstrap values and closely matched known mammalian evolutionary relationships, with minor discrepancies.

### Codon-based positive selection analyses

Before performing positive selection tests, we examined the sequences for recombination events since recombination might lead to inaccurate positive outcomes. The maximum likelihood method was employed to study molecular evolution. Nucleotide sequences encoding proteins can help identify evolutionary events involving episodic or persistent positive selection. Positive selection processes were tested during the molecular evolution of genes in the GBP5, GZMB, IFNG, IRF7, KLRD1, RTP4, TNFSF4, and TRAT1 gene clusters. We utilized the CODEML tool to obtain log-likelihood function values for site models M8 and M8a. We conducted a likelihood ratio test (LRT) to identify sites experiencing positive selection pressure (ω > 1) across all branches of the mammalian evolutionary tree. There was statistical significance with an LRT value of 175.19 and a p-value of 0.01. By merging the sequences of the GBP5, GZMB, IFNG, IRF7, KLRD1, RTP4, TNFSF4, and TRAT1 genes, in silico studies demonstrated that every node of the mammalian evolutionary tree contains areas subject to positive selection. After that, we found the locations using the Bayes empirical Bayes (BEB) method. During their evolution, sites were deemed to have been subject to positive selection if their posterior probability (PP) was greater than 0.9. All branches of the evolutionary tree point to the same amino acid sites that are subject to positive selection and these places have been given probabilities and values. Most possible sites were identified inside the conserved domain regions of the proteins in the cluster (Fig 1).

We observed widespread positive selection events in these genes’ molecular history and explored the potential impact of episodic positive selection on the genes’ molecular evolution. Positive selection typically happens by influencing particular sites within specific clades and branches of a phylogenetic tree. After calculating the log-likelihood values for two branch-site models, we conducted Likelihood Ratio Tests (LRT) on specific clades and branches of the mammalian phylogenetic tree under strict and lenient conditions. To investigate whether certain sites are under positive selection (ω > 1) or relaxed negative selection in specific branches (foreground branches) of the mammalian phylogeny compared to other branches, we initially used the branch-site test 1 (Zhang et al., 2005). Multiple verified phylogenetic branches and clades exhibited statistically significant likelihood ratio test (LRT) values. Under stringent criteria, selection events were identified in 20 test branches, but under lenient conditions, they were recognized in 27 test branches (Table 1). Even in lenient testing conditions, the likelihood ratio test (LRT) scores for the branches and clades of the phylogenetic tree were not statistically significant. We did not find any relaxed negative or positive selection for these branches.

**Table 1:** Detailed site-by-site results from the FEL analysis.

Gene	Codon	Synonymous substitution rate (α)	Non-synonymous substitution rate (β)	(α = β)	LRT	p-value	Total branch length	class
GBP5	5	0	1.69	0.968	7.776	0.0053	2.731	Diversifying
	6	0	1.926	1.376	4.873	0.0273	3.884	Diversifying
	11	0	1.322	0.989	3.856	0.0496	2.793	Diversifying
	35	0	0.885	0.577	4.096	0.043	1.63	Diversifying
	162	0.38	2.981	1.937	5.871	0.0154	5.467	Diversifying
	215	0	1.642	1.11	4.856	0.0276	3.133	Diversifying
	316	0	1.117	0.691	5.56	0.0184	1.951	Diversifying
	421	0	1.445	1.029	4.402	0.0359	2.905	Diversifying
	435	0	1.575	1.129	5.272	0.0217	3.188	Diversifying
	446	0	1.281	0.834	5.359	0.0206	2.353	Diversifying
GZMB	75	0	0.983	0.723	3.012	0.0826	1.952	Diversifying
	130	0	0.687	0.456	3.211	0.0731	1.23	Diversifying
	132	0	0.823	0.576	2.811	0.0936	1.555	Diversifying
	170	0	1.288	0.913	3.846	0.0499	2.463	Diversifying
	172	0	1.002	0.728	3.047	0.0809	1.965	Diversifying
	197	0	2.797	1.915	5.445	0.0196	5.168	Diversifying
	215	0	2.9	1.964	6.379	0.0115	5.3	Diversifying
	223	0	1.901	1.321	4.702	0.0301	3.564	Diversifying
	237	0.54	4.06	2.613	4.7	0.0302	7.051	Diversifying
	260	0	7.571	2.842	10.49	0.0012	7.669	Diversifying
	281	0	0.841	0.576	3.523	0.0605	1.555	Diversifying
IFNG	21	0	1.194	0.783	5.309	0.0212	2.876	Diversifying
	54	0	0.753	0.528	3.765	0.0523	1.94	Diversifying
	99	0	1.096	0.743	4.438	0.0351	2.731	Diversifying
	116	0.49	2.563	1.634	3.617	0.0572	6.006	Diversifying
	120	0.07	4.329	2.532	4.269	0.0388	9.306	Diversifying
IRF7	148	0	2.503	0.925	3.283	0.07	6.233	Diversifying
	161	0	2.295	1.421	3.074	0.0796	9.571	Diversifying
	179	0.09	2.456	1.742	3.37	0.0664	11.74	Diversifying
	181	0.15	1.093	0.648	3.67	0.0554	4.368	Diversifying
	253	0.07	2.787	1.1	2.921	0.0874	7.409	Diversifying
	370	0	0.471	0.323	3.1	0.0783	2.175	Diversifying
	474	0	1.294	0.412	11.68	0.0006	2.775	Diversifying
Klrd1	158	0.08	1.39	0.918	3.311	0.0688	5.077	Diversifying
	189	0	0.942	0.644	3.101	0.0782	3.558	Diversifying
	225	0	0.875	0.635	4.334	0.0374	3.508	Diversifying
	235	0.32	1.809	1.207	3.914	0.0479	6.67	Diversifying
	236	0	0.637	0.514	2.986	0.084	2.841	Diversifying
	252	0.15	0.677	0.468	2.725	0.0988	2.586	Diversifying
	268	0	3.421	2.215	5.524	0.0188	12.24	Diversifying
	270	0.24	1.801	1.405	4.09	0.0431	7.764	Diversifying
	272	0	3.33	2.213	7.117	0.0076	12.23	Diversifying
	274	0	1.177	0.797	6.915	0.0085	4.408	Diversifying
	276	0	0.966	0.682	6.166	0.013	3.768	Diversifying
	289	0	0.699	0.561	3.249	0.0715	3.102	Diversifying
	290	0.34	2.069	1.336	5.638	0.0176	7.384	Diversifying
	292	0.06	2.599	1.503	7.414	0.0065	8.308	Diversifying
	304	0	1.012	0.722	3.563	0.0591	3.99	Diversifying
Rtp4	25	0	1.342	0.792	2.842	0.0919	2.409	Diversifying
	53	0	1.731	1.075	5.869	0.0154	3.267	Diversifying
	79	0	0.753	0.498	3.14	0.0764	1.514	Diversifying
	85	0	1.043	0.766	2.946	0.0861	2.328	Diversifying
	109	0	2.056	1.188	3.257	0.0711	3.611	Diversifying
	125	0	1.369	0.92	3.1	0.0783	2.798	Diversifying
	151	0	1.04	0.771	3.073	0.0796	2.343	Diversifying
	152	0	0.622	0.424	3.04	0.0813	1.289	Diversifying
	173	0.6	3.197	1.97	2.799	0.0944	5.988	Diversifying
	184	0	2.512	1.525	8.134	0.0043	4.636	Diversifying
	195	0	2.839	1.525	9.264	0.0023	4.635	Diversifying
	196	0.07	3.725	2.159	3.891	0.0485	6.562	Diversifying
	199	0	1.754	1.166	3.689	0.0548	3.545	Diversifying
	208	0	1.088	0.829	3.178	0.0746	2.521	Diversifying
	235	0	3.386	2.776	3.551	0.0595	8.438	Diversifying
	249	0	4.603	2.443	4.928	0.0264	7.426	Diversifying
	261	0	1.882	1.226	3.569	0.0589	3.725	Diversifying
	282	0	1.833	1.101	2.833	0.0923	3.348	Diversifying
	393	0	1.843	1.18	2.729	0.0985	3.586	Diversifying
	403	0	1.823	1.029	2.932	0.0869	3.127	Diversifying
	406	0	3.058	1.953	3.004	0.0831	5.937	Diversifying
	409	0	8.143	7.189	3.489	0.0618	21.85	Diversifying
	470	0	9.818	3.175	2.895	0.0888	9.651	Diversifying
	479	0	22.38	18.44	4.826	0.028	56.06	Diversifying
	570	0	1.937	1.298	2.82	0.0931	3.947	Diversifying
	573	0	1.778	0.984	3.196	0.0738	2.991	Diversifying
	578	0	1.92	1.075	2.932	0.0868	3.267	Diversifying
	582	0	4.18	2.174	4.207	0.0402	6.607	Diversifying
	595	0.71	3.848	2.784	2.828	0.0926	8.461	Diversifying
	611	0	2.891	1.571	4.372	0.0365	4.774	Diversifying
TNFSF4	19	0.43	1.408	0.984	2.793	0.0947	7.414	Diversifying
	64	0.25	1.372	1.002	3.629	0.0568	7.546	Diversifying
	158	0.3	1.67	1.239	3.188	0.0742	9.331	Diversifying
	223	0	0.449	0.332	2.822	0.093	2.498	Diversifying
	235	0	0.877	0.684	5.097	0.024	5.152	Diversifying
TRAT1	12	0	1.471	0.91	4.67	0.0307	2.959	Diversifying
	21	0	0.492	0.299	2.895	0.0889	0.971	Diversifying
	24	0	0.671	0.454	3.187	0.0742	1.475	Diversifying
	58	0	0.862	0.651	2.756	0.0969	2.116	Diversifying
	93	0	1.341	1.05	3.21	0.0732	3.413	Diversifying
	118	0	0.804	0.55	3.787	0.0516	1.786	Diversifying
	131	0	0.451	0.282	2.736	0.0981	0.917	Diversifying

**Fig 2:**
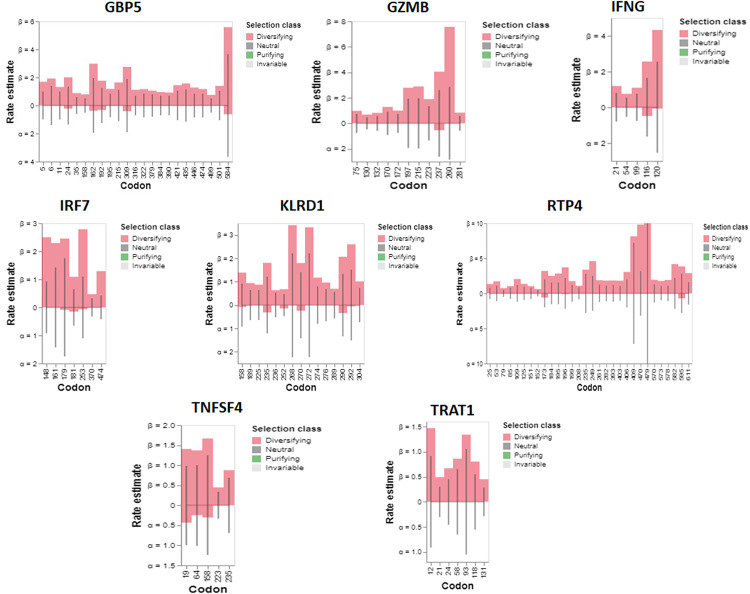
Results for synonymous (α) and non-synonymous (β) rates at each site are displayed as bars, representing maximum probability estimates. The line under the null model (α = β) shows the estimations. This value censors estimates that are more than 10.

Test 1 could not distinguish between positive selection and relaxation of selective constraint, so we utilized test 2, developed by the authors, to directly assess the presence of positive selection in the lineages of interest. We tested the hypothesis that certain branches or groups of branches in the phylogenetic tree are under positive selection pressure (ω > 1) compared to other branches (A1 vs. A) for the branches and clades that passed test 1 (Zhang et al., 2005). We identified positive selection events in 15 of 20 branches using strict criteria and 26 out of 27 under less rigorous conditions (Table 1). An in silico investigation of the molecular evolution of the GBP5, GZMB, IFNG, IRF7, KLRD1, RTP4, TNFSF4, and TRAT1 genes (Fig 2) revealed independent positive selection events in most branches of the mammalian phylogenetic tree.

The Bayes Empirical Bayes (BEB) method identified several codons under positive selection (PP > 0.95) for each gene. To interpret the functional potential of these evolutionary signatures, we mapped the positively selected sites onto known functional protein domains (S2 Table). Notably, many selected sites were located within critical functional domains: for example, sites in GBP5 were found within its GTPase domain, suggesting adaptation in its core enzymatic and oligomerization functions, while selected sites in GZMB clustered in the serine protease active site region, potentially influencing its substrate specificity and catalytic efficiency during cytotoxic immune responses.

**Fig 3:**
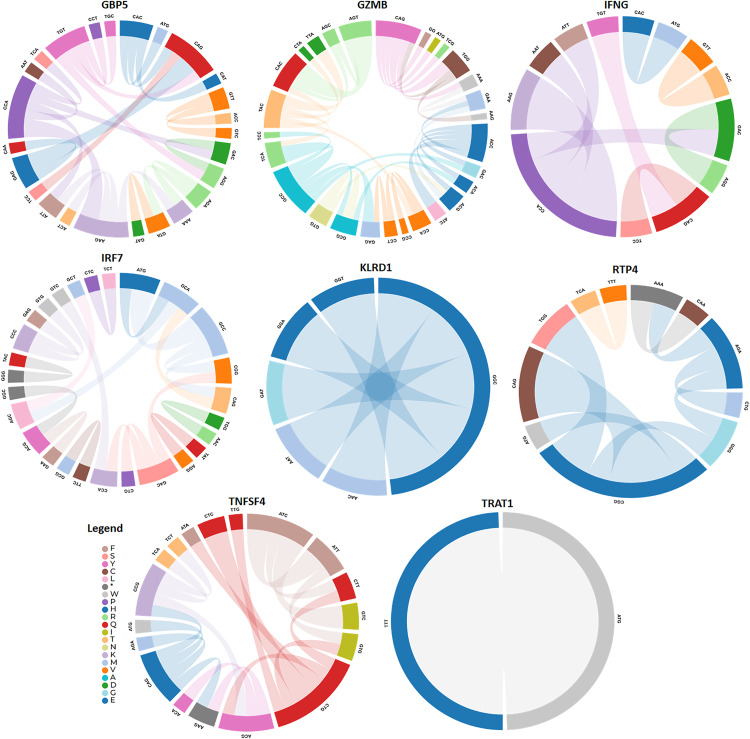
Three-hit replacements frequently occur in non-synonymous substitutions. Three-hit replacements with 3H+ support are substitutions at sites with an ER (3H + :2H) configuration. Three-hit substitutions with a 2H but not 3H+ support are replacements that happen at locations where the ratio of ER (3H + :2H) is less than 1 and the ratio of ER (2H: 1H) is not specified. The histogram displays the branch lengths where the two substitutions are estimated to occur.

The increased favourable selection rates on these sequences may be due to dS saturation or inadequate taxon sampling, impacting the reconstruction of the ancestral sequence and the calculation of several model parameters. It is widely known that this problem can sometimes yield inaccurately positive outcomes. The positions in the primary protein structure of the genes GBP5, GZMB, IFNG, IRF7, KLRD1, RTP4, TNFSF4, and TRAT1 were identified using the corresponding Figs and table (Fig 3). Closely related populations and species of animals appear to have had rapid evolutionary repercussions triggered by their microbiota, and these effects may have even impacted the recent evolution of humans. The gut microbiota has helped in the adaptive evolution of mammalian gut shapes designed to accommodate helpful microbes. The gut microbiota probably contributed to the development of both innate and adaptive immune systems in mammals.

### Adaptation selection analysis

**Fig 4:**
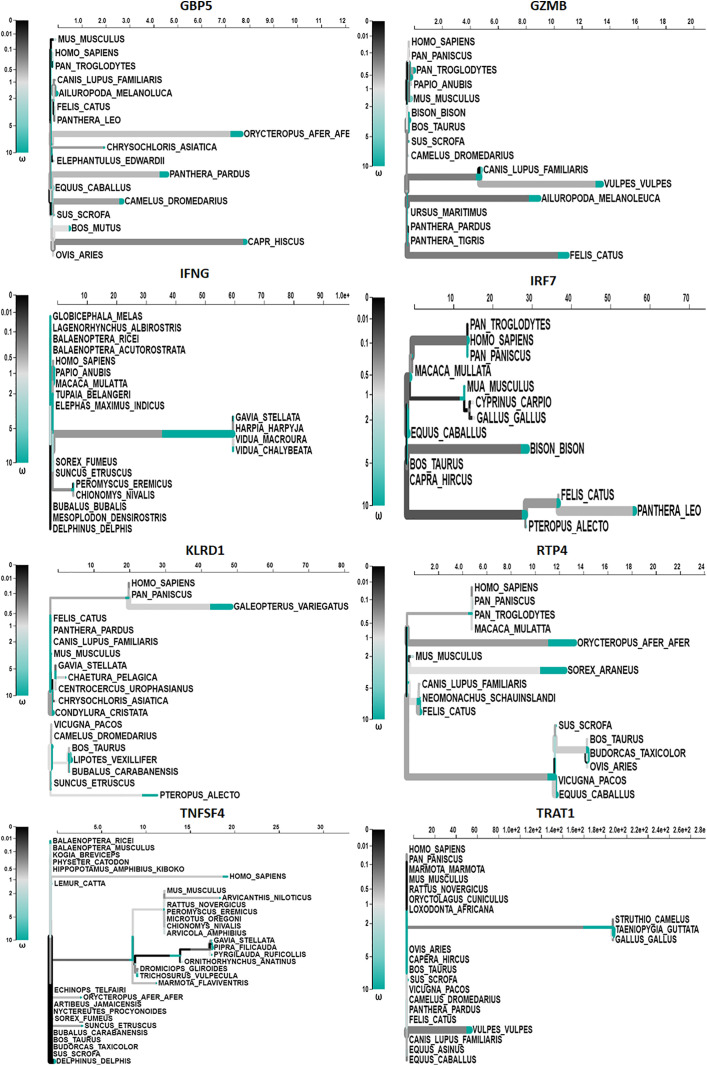
An aBSREL adaptive model tree was applied to analyze the full-length GBP5, GZMB, IFNG, IRF7, KLRD1, RTP4, TNFSF4, and TRAT1 genes across mammalian species. The inferred ω distribution determines the shade of the branches. Alleles determined to be under positive selection, with a statistical significance of less than 0.05 after adjustment, are visually represented by thick black branches.

aBSREL detected evidence of episodic diversifying selection on two out of the 35 branches in the GBP5 phylogeny. A total of 35 branches underwent official testing to diversify their selection. The significance of the results was evaluated using the Likelihood Ratio Test at a significance level of p < 0.05 after adjusting for multiple comparisons (Fig 4). The comprehensive findings table provides information on the significance and number of rate categories inferred at each branch (Table 1). aBSREL detected episodic diversifying selection on 9 out of 29 branches in the GZMB phylogeny (Fig 4); 29 branches underwent formal testing for diversifying selection. The significance was evaluated using the Likelihood Ratio Test at a threshold of p < 0.05, following adjustment for multiple testing. The full findings table contains information about the significance and number of rate categories inferred at each branch.

**Fig 5:**
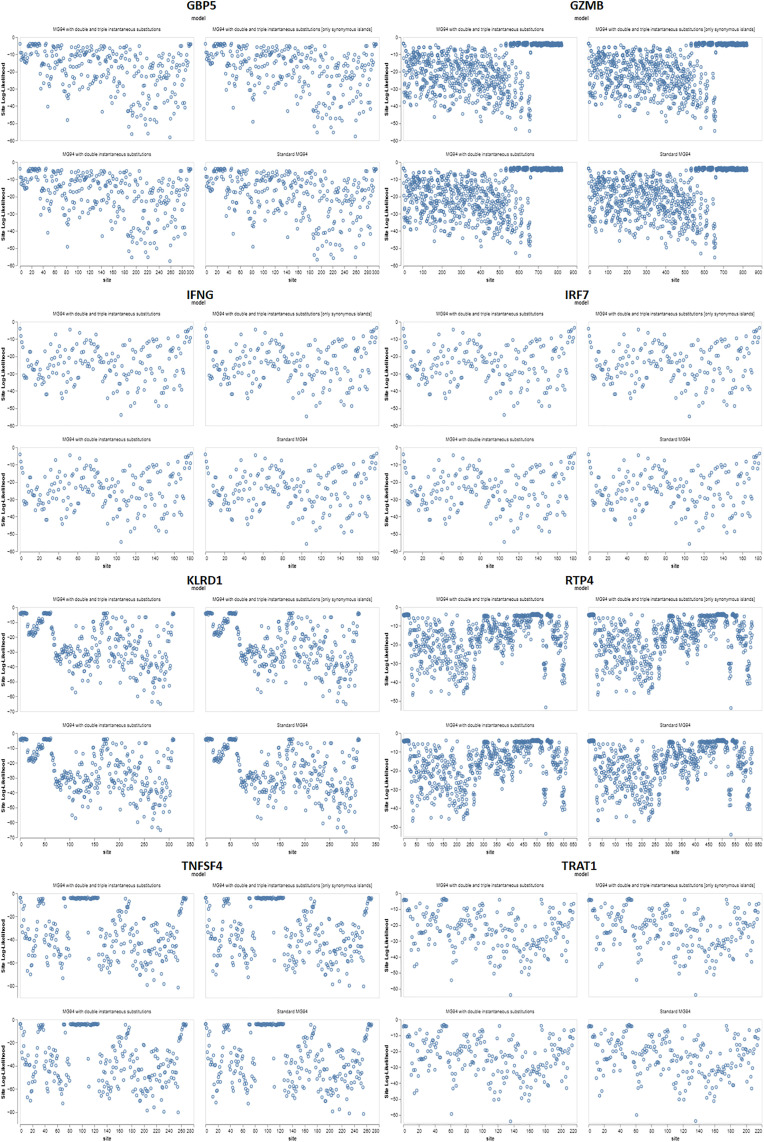
The Site Log-Likelihood analyses provide estimated ω rate distributions for the relative rate distribution (mean 1) for site-to-site non-synonymous rate variation that fits MG94 with double and triple instantaneous substitutions.

aBSREL detected episodic diversifying selection on two branches out of 35 branches in the IFNG phylogeny (Fig 4). Thirty-five branches underwent official testing for diversifying selection. The significance of the results was evaluated using the Likelihood Ratio Test at a significance level of p < 0.05, with adjustments made for multiple testing (Fig 5). The comprehensive findings table contains information on the significance and number of rate categories inferred at each branch (Table 1). aBSREL detected episodic diversifying selection on 7 out of the 24 branches in the IRF7 phylogeny (Fig 4). 24 branches underwent official testing for diversifying selection. The significance of the results was evaluated using the Likelihood Ratio Test at a significance level of p < 0.05 after adjusting for multiple comparisons. The comprehensive findings table provides information on the significance and number of rate categories inferred at each branch (Fig 4). aBSREL detected evidence of episodic diversifying selection on four out of 35 branches in the KLRD1 phylogeny (Fig 4). A total of 35 branches underwent official testing to diversify their selection. The significance of the results was evaluated using the Likelihood Ratio Test at a threshold of p < 0.05, with adjustments made for multiple testing. The full findings table contains information about the significance and number of rate categories inferred at each branch. aBSREL detected episodic diversifying selection on 8 of the 29 branches in the RTP4 phylogeny (Fig 4). 29 branches underwent formal testing for diversifying selection. The significance was evaluated using the Likelihood Ratio Test at a threshold of p < 0.05, following adjustment for multiple testing. The full findings table contains information about the significance and number of rate categories inferred at each branch. aBSREL detected episodic diversifying selection on two out of the 58 branches in the TNFSF4 phylogeny (Fig 4). A total of 58 branches underwent formal testing to assess the presence of diversifying selection. After adjusting for multiple tests, the significance was evaluated using the Likelihood Ratio Test with a p < 0.05 threshold. The comprehensive findings table provides information on the significance and number of rate categories inferred at each branch. aBSREL detected evidence of episodic diversifying selection on two out of the 41 branches in the TRAT1 phylogeny (Fig 4). Forty-one branches underwent formal testing for a diverse selection. The significance of the results was evaluated using the Likelihood Ratio Test at a significance level of p < 0.05 while accounting for multiple testing (Fig 5). The full findings table contains information about the significance and number of rate categories inferred at each branch (Table 1).

### Recombination analysis

**Fig 6:**
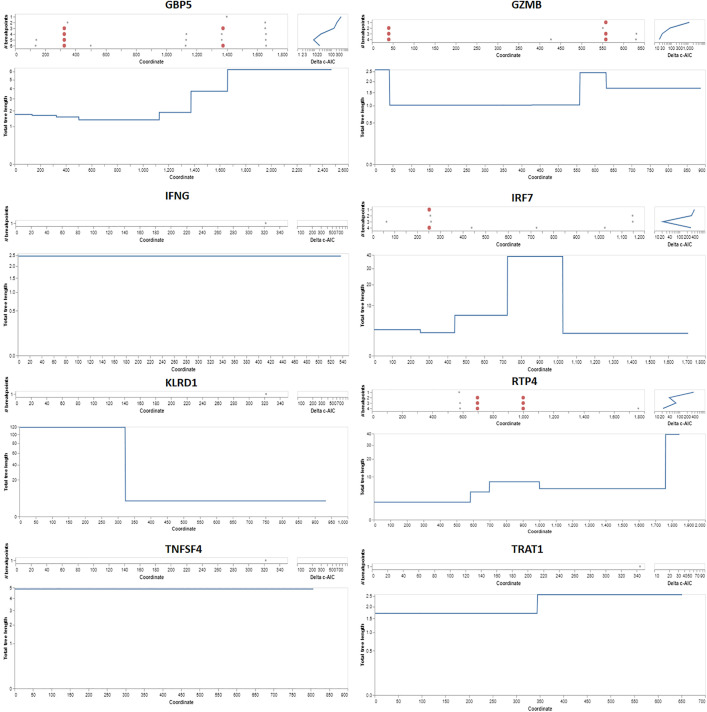
Left: the algorithm’s best estimate of where to put breakpoints for each number of breakpoints considered. Correct: the increase in the c-AIC score (log scale) when breakpoint numbers increase.

The GARD analysis detected recombination breakpoints in the GBP5 gene. GARD analyzed a total of 13,556 models at a speed of 21.42 models per second. The alignment consisted of 1183 possible breakpoints, resulting in a search space of 635810244030937500 models with a maximum of 7 breakpoints. However, the genetic algorithm only searched 0.00% of this search space (Fig 6). The AICc score of the best-fitting GARD model, which permits different topologies between segments (29983.2), is compared to that of the model assuming the same tree for all partitions inferred by GARD but allowing different branch lengths between partitions (30120.2). This suggests that the multiple-tree model may be preferred over the single-tree model by an evidence ratio of 100 or greater. This indicates that at least one of the breakpoints represents a genuine topological incongruence. The GARD analysis detected recombination breakpoints within the GZMB gene. GARD analyzed a total of 9905 models at a speed of 50.54 models per second. The alignment consisted of 458 possible breakpoints, resulting in a search space of 166123556333 models with a maximum of 5 breakpoints. However, the genetic algorithm only searched 0.00% of this search space (Fig 6).

The AICc score of the best-fitting GARD model, which permits different topologies between segments (10967.6), is compared to that of the model assuming the same tree for all partitions inferred by GARD but allowing different branch lengths between partitions (11723.4). This suggests that the multiple-tree model may be preferred over the single-tree model by an evidence ratio of 100 or greater. This indicates that at least one of the breakpoints represents a genuine topological incongruence. GARD did not detect any signs of recombination in IFNG. GARD analyzed a total of 2630 models at a speed of 49.62 models per second. The alignment consisted of 409 possible breakpoints, resulting in a search space of 409 models with a maximum of 1 breakpoint (Fig 6). The genetic algorithm examined 643.03% of this search space. The comparison of the AICc scores between the best-fitting GARD model, which permits different topologies between segments (9309.1), and the model that assumes the same tree for all partitions inferred by GARD but allows different branch lengths between partitions (9309.1), indicates that the multiple tree model cannot be favored over the single tree model by an evidence ratio of 100 or more. This suggests that some or all of the breakpoints may indicate rate variation rather than topological incongruence. Notably, GARD detected evidence of recombination breakpoints in the IRF7 and KLRD1 genes. GARD analyzed a total of 12,831 models at a speed of 41.52 models per second. The alignment consisted of 1253 possible breakpoints, resulting in a search space of 25635663809007 models with a maximum of 5 breakpoints (Fig 6). However, the genetic algorithm only searched 0.00% of this search space. The AICc score of the best-fitting GARD model, which permits different topologies between segments (23104.9), is compared to that of the model assuming the same tree for all partitions inferred by GARD but allowing different branch lengths between partitions (23164.6). This suggests that the multiple tree model may be preferred over the single tree model by an evidence ratio of 100 or more, indicating that at least one of the breakpoints represents a genuine topological incongruence. GARD analyzed a total of 1419 models at a speed of 43.00 models per second. The alignment consisted of 717 possible breakpoints, resulting in a search space of 257,403 models that might have up to 2 breakpoints (Fig 6).

The genetic algorithm examined only 0.55% of this search area. An evidence ratio of 100 or more favors the multiple tree model over the single tree model is shown by comparing the AICc scores of the best-fitting GARD model (16444.6) and the model that assumes the same tree for all partitions inferred by GARD (16511.0), which allows different branch lengths between partitions. This points to the possibility that a real topological incongruence is represented by one of the breakpoints. Finding recombination breakpoints in the RTP4 gene was accomplished by the Genetic Algorithm for Recombination Detection (GARD). GARD processed 11,981 models at a rate of 19.45 per second. The alignment consisted of 1041 possible breakpoints, resulting in a search space of 10,138,915,336,889 models with a maximum of 5 breakpoints. However, the genetic algorithm only examined 0.00% of this search space. The Genetic Analysis and Recombination Detection (GARD) method did not detect any evidence of recombination in the TNFSF4 gene.

GARD analyzed 1793 models at a speed of 23.29 models per second. The alignment consisted of 559 possible breakpoints, resulting in a search space of 559 models with a maximum of 1 breakpoint (Fig 6). The genetic algorithm examined 320.75% of this search area. The GARD analysis detected recombination breakpoints in the TRAT1 gene. GARD analyzed 2298 models at a speed of 69.64 models per second. The alignment consisted of 442 possible breakpoints, resulting in a search space of 97903 models with a maximum of 2 breakpoints. The genetic algorithm searched 2.35% of this search area.

### Functional analysis

Initially, we identified all statistically enriched terms, such as GO/KEGG terms, canonical pathways, and hallmark gene sets. This was done based on either the default choices under Express Analysis or those made during Custom Analysis. We then calculated accumulative hypergeometric p-values and enrichment factors used for filtering. The remaining important phrases were further organized into a hierarchical tree structure using Kappa-statistical similarities among their gene memberships, similar to the approach employed in the NCI DAVID site. A threshold of 0.3 kappa score was used to convert the tree into term clusters. The terms contained in each cluster are exported in the Excel worksheet titled “Enrichment Analysis.” We extracted a subset of key phrases from the entire cluster and transformed them into a network layout. Each word is depicted as a circular node, sized according to the number of input genes associated with that term. The node’s color indicates its cluster identification, meaning nodes of the same color belong to the same cluster. Terms with a similarity score greater than 0.3 are connected by an edge, with the thickness of the edge representing the similarity score.

The network is shown using Cytoscape, with a “force-directed” structure and edge bundling to enhance clarity. Positively chosen sites have been found in the GBP5 protein, which contains the Guanylate-binding protein (GBP) and the N-terminal domain of Interferon (IFN)-inducible GTPase. These pathogens are a wide variety of bacteria, viruses, and protozoa, playing significant roles in innate immunity against them. After infection, it is drawn to bacteria that have escaped from vacuoles or contain pathogens, and it functions as a positive regulator of the assembly of inflammasomes by encouraging the release of ligands from the bacteria. This releases ligands that are recognized by inflammasomes, such as double-stranded DNA (dsDNA), that activates the AIM2 inflammasome or lipopolysaccharide (LPS), which activates the non-canonical CASP4/CASP11 inflammasome.

The GZMB protein has a trypsin-like serine protease domain that contains the active site and is found in members of the trypsin family. The serine proteases from the trypsin family exhibit catalytic activity through a charge relay system. This system involves an aspartic acid residue that forms a hydrogen bond with a histidine, forming a hydrogen bond with a serine. The IFNG protein, which possesses the IFN-gamma domain, exhibits antiviral properties and regulates the immune system. This substance is highly effective at stimulating macrophages and can inhibit altered cell growth. It can enhance the antiviral and anticancer effects of type I interferons. The interferon-regulatory factor 7 includes the truncated CREB-binding protein domain. The DRAF1 (double-stranded RNA-activated factor 1) consists of these two subunits (Fig 8). The production of viral double-stranded RNA (dsRNA) during viral transcription or replication results in the activation of DRAF1. The DNA-binding specificity of DRAF1 is directly related to the transcriptional stimulation of ISGs (interferon-alpha, beta-stimulated genes). The protein IRF-3 is initially present in the cytoplasm of cells that have not been infected, but it moves to the nucleus after a viral infection occurs.

**Fig 7:**
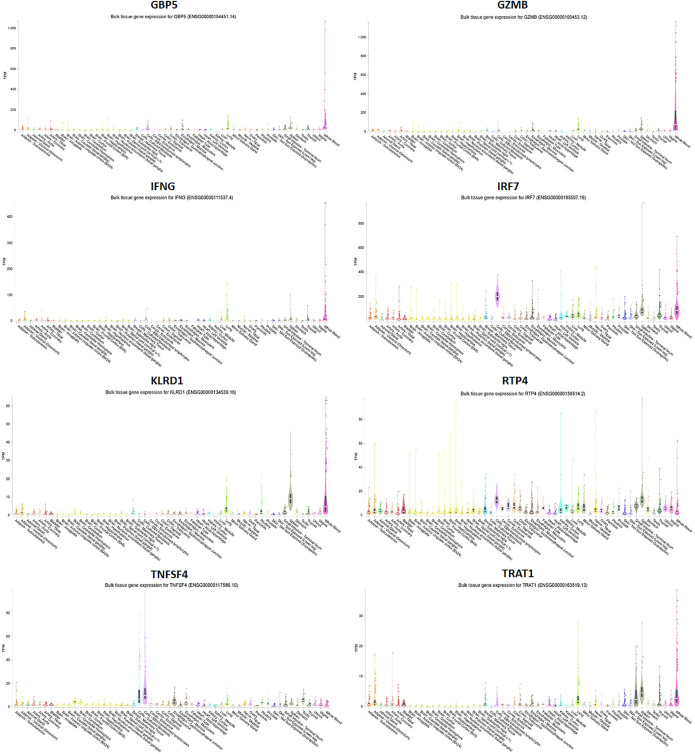
Tissue-specific expression of GBP5, GZMB, IFNG, IRF7, KLRD1, RTP4, TNFSF4, and TRAT1 genes in humans used as a reference genome. The expression data of these genes were revealed across various tissues courtesy of the GTEx consortium.

**Fig 8:**
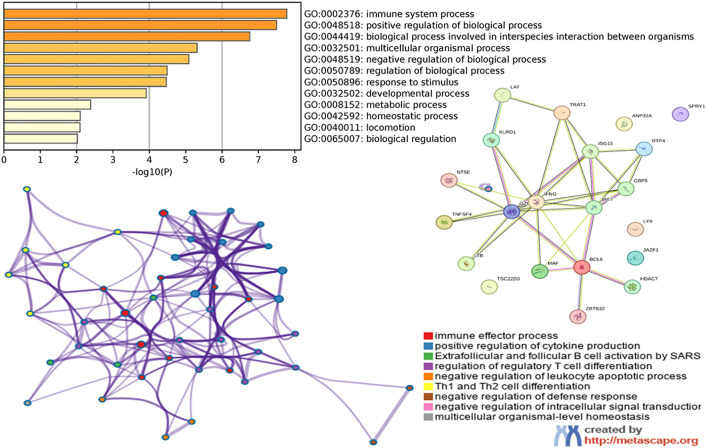
Detected all significantly enriched terms, including GO/KEGG terms, canonical pathways, and gene sets for GBP5, GZMB, IFNG, IRF7, KLRD1, RTP4, TNFSF4, and TRAT1. Selected key phrases from the entire cluster and transformed them into a network arrangement. A protein-protein network was built by extracting connections among the genes GBP5, GZMB, IFNG, IRF7, KLRD1, RTP4, TNFSF4, and TRAT1 from a data source of protein-protein interactions.

**Fig 9:**
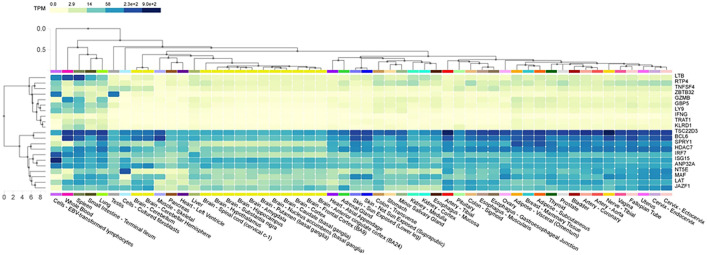
Expression analysis of GBP5, GZMB, IFNG, IRF7, KLRD1, RTP4, TNFSF4, and TRAT1 proteins in tissues.

The translocation of IRF-3 is accompanied by an elevation in the phosphorylation of serine and threonine residues, and its interaction with the CREB co-activator only occurs following infection. The carbohydrate-recognition domain (CRD), often called the C-type lectin domain (CTL), comprises around 110–130 amino acid residues. Two disulfide connections are formed by four cysteines, all of which are fully conserved. Lectins have a very diverse range of structural characteristics and functional attributes. The capacity to bind carbohydrates may have independently and sporadically developed in several unrelated families, each producing a conserved structure for a distinct purpose. Animal lectins function as immune system recognition molecules and are involved in cell migration, pathogen protection, immunological regulation, and the avoidance of autoimmunity (Fig 8). TNF is the name of a domain found in the protein known as tumor necrosis factor. Families of cytokines can assemble into complexes with three subunits that are the same or distinct. The mature T-cell receptor uses TNF to induce apoptosis, and the p75 TNF receptor mediates this process. An essential tool found in the GTEx database is an expression quantitative trait locus (eQTL) browser. This browser functions as a storage and graphical display of data collected from a nationwide research initiative that sought to discover links between genetic variants and high-throughput molecular-level expression phenotypes (Fig 9). It is worth mentioning that a considerable number of genes display connections with different tissues. Gbp5, Gzmb, Ifng, Irf7, Klrd1, Rtp4, and Trat1 genes exhibit significant expression in whole blood, whereas Klrd1, Rtp4, and Trat1 genes revealed expression in the spleen (Fig 7). Tnfsf4 and Rtp4 have shown expression in lymphocytes. Nevertheless, our examination of the mean expression levels of all (significant) genes using various enrichment techniques yielded inconclusive results about the tissues expected to have a higher prevalence of diseases and well-established biological processes (Table 2).

**Table 2:** The network performed GO enrichment analysis to identify the underlying “biological meanings”.

GO	Category	Description	Count	%	Log10(P)	Log10(q)
GO:0002252	GO Biological Processes	immune effector process	7	33.33	−7.78	−3.46
GO:0001819	GO Biological Processes	positive regulation of cytokine production	7	33.33	−7.50	−3.46
WP5218	WikiPathways	Extrafollicular and follicular B cell activation by SARS CoV 2	4	19.05	−6.67	−3.11
GO:0045589	GO Biological Processes	regulation of regulatory T cell differentiation	3	14.29	−5.69	−2.50
GO:2000107	GO Biological Processes	negative regulation of leukocyte apoptotic process	3	14.29	−5.09	−2.07
hsa04658	KEGG Pathway	Th1 and Th2 cell differentiation	3	14.29	−4.46	−1.72
GO:0031348	GO Biological Processes	negative regulation of defense response	3	14.29	−3.00	−0.56
GO:1902532	GO Biological Processes	negative regulation of intracellular signal transduction	3	14.29	−2.13	0.00
GO:0048871	GO Biological Processes	multicellular organismal-level homeostasis	3	14.29	−2.10	0.00

## Discussion

The gut mucosal immune system interfaces the internal body and the external environment. The microorganisms present in the gut environment continuously impact the immune system. In return, the host’s immune system influences the microbiome’s makeup [67]. The widespread and persistent positive selection observed across the eight immune genes (GBP5, GZMB, IFNG, IRF7, KLRD1, RTP4, TNFSF4, and TRAT1) strongly suggests an ongoing evolutionary arms race with a diverse array of pathogens. The specific functional roles of these genes allow us to hypothesize the classes of pathogens that have likely exerted the strongest selective pressures [68]. We analyzed a group of 8 protein-coding orthologs (GBP5, GZMB, IFNG, IRF7, KLRD1, RTP4, TNFSF4, and TRAT1) that are present in the genomes of humans, monkeys, dogs, cats, cows, mice, and domestic yaks. Our goal was to identify signs of positive selection in these genes. Within these genes exhibiting statistically significant signals (P < 0.05 corrected), further analysis of branch sites indicated the presence of positive selection, specifically along the mammalian lineages. The M8 model, which employs positive selection, was utilized to detect variations at the codon level. A Markov Chain Monte Carlo (MCMC) model, implemented in MrBayes on the Selecton server, was utilized to ascertain the disparity at the codon level. Values were calculated for each place in both cases. The results of our study demonstrate that the coding sequences of eight genes exhibit domain conservation when analyzed using MAFFT protein alignments (Fig 1). These findings indicate that non-identical protein switches in areas undergoing purifying selection are detrimental to health and, therefore, unlikely to become established during evolution. The significant positive selection in GBP5, a key activator of inflammasomes against cytosolic bacteria (e.g., Listeria, Salmonella) and a regulator of antiviral responses, points to intense pressure from intracellular bacteria and viruses [68]. Pathogens that escape phagosomes or inject effectors to manipulate host cell machinery would create a strong selective advantage for mutations in GBP5 that enhance detection or circumvent pathogen inhibition [69]. Similarly, the selection in IRF7 and IFNG, central orchestrators of the antiviral interferon response, implicates a history of conflict with viruses. RNA viruses, with their high mutation rates and rapid evolution, are particularly potent drivers of such adaptation, constantly presenting new ligands and decoys that select for counter-adaptations in the host’s interferon signaling pathway.

Genetic transfers and duplications are fundamental in developing all major adaptive immune molecular systems on a horizontal scale [69]. Prior research on the evolution of immune genes in birds mostly examined the co-evolution of disease hotspots, such as MAVS, in the context of influenza virus infection. They play a role in activating lymphocytes, regulating the immune system, stimulating T regulatory cells, and influencing the development and tolerance of autoimmunity [53]. The impact of selection on host organisms in regulating gut microbiota during the adaptive evolution of mammalian species remains inadequately understood despite the intricate mechanisms developed by our ancestors’ predecessors. Immune genes in mammalian genomes are evolving rapidly, suggesting the presence of pathogens and co-evolutionary dynamics known as red-queen dynamics [70]. The extent to which genetic variation in immune genes is affected by differences in gut microbiota among mammalian species remains uncertain. Bacteria can change rapidly during an evolutionary arms race, making it difficult for mammalian hosts to constantly adapt to control the microbiota, which also evolves to compete among itself [71]. Multiple verified phylogenetic branches and clades exhibited statistically significant likelihood ratio test (LRT) values. Under stringent criteria, selection events were identified in 20 test branches, but under lenient conditions, they were recognized in 27 test branches (Table 1). Even in lenient testing conditions, the likelihood ratio test (LRT) scores for the branches and clades of the phylogenetic tree were not statistically significant. We did not find evidence of relaxed negative or positive selection for these branches. Test 1 could not distinguish between positive selection and relaxation of selective constraint, so we utilized test 2, developed by the authors, to directly assess the presence of positive selection in the lineages of interest. We tested the hypothesis that certain branches or groups of branches in the phylogenetic tree are under positive selection pressure (ω > 1) compared to other branches (M2a vs. M2) for the branches and clades that passed test 1 [72]. The adaptive evolution in TNFSF4 (OX40L) and TRAT1, both involved in fine-tuning T-cell receptor signaling and co-stimulation, likely reflects a struggle to optimize the adaptive immune response against a broad spectrum of challenges. This could include pressure from persistent pathogens that exhaust T cells (e.g., chronic viruses like HIV or HCV) or from pathogens that manipulate co-stimulatory pathways to evade immunity [10,11]. Selection on these genes would favor variants that strengthen effective T-cell responses while potentially limiting immunopathology or countering pathogen-derived immunosuppressive factors

The functional repertoires in mammalian gut microbiotas have likely supported the evolution and diversification of chitin-eating and herbivory, the specialization of mammalian species and communities on hazardous diets, and potentially even recent dietary changes in human evolution [73]. Furthermore, there is increasing evidence that animals have adapted to depend on signals from the specific gut bacteria of their hosts during postnatal development and functioning. House mice colonized with the gut microbiota of rats or humans did not exhibit fully differentiated T cell repertoires, unlike those colonized with the gut microbiota of other house mice [74]. Mammals have evolved to rely on the specific gut microbiota of their hosts for guidance during postnatal growth and function, as supported by a growing body of research. These findings suggest that the immunological development of house mice has evolved to include components of their particular gut flora since the divergence of mice and rats [75]. The adaptation of a species to a new food is a significant driving force in the evolution of that species. The dietary modifications that occurred throughout the development of several primate species, including humans, have been extensively recorded across time [73]. The investigation also covers the extent of taxonomic conservation of specific immune-modulatory mechanisms, including the synthesis of antimicrobial peptides and the function of microbial metabolites. We have emphasized data indicating that, whereas certain systems are preserved, others may be more specialized and represent responses to certain ecological niches or evolutionary forces. We have found both distinct and maybe universal patterns in the interactions between the immune system and gut bacteria in several species. For example, the need for gut microbiota to support immune system maturation seems to be a universal feature across taxa, even though the precise microbial species and immunological pathways implicated may vary.

Additionally, some genes have been identified as being involved in positive selection driven by nutrition. An extensively researched instance can be observed in pancreatic ribonuclease in old-world monkey species [41]. Due to the monkey’s dietary changes, the protein in this species has developed an enhanced capacity to break down bacterial DNA. Another instance is lysozyme, which facilitates the breakdown of intestinal microorganisms. This protein has demonstrated positive selection in various primate groups, including humans [76]. The langur monkey, a species that has developed a foregut fermentation mechanism of digesting comparable to ruminants, provides the clearest understanding of the nature of this selection [77]. However, it has been hypothesized that the transition to a diet mostly consisting of meat, which would have necessitated adaptations in bacterial digestion, may have been a contributing factor [73]. Studies of alanine-glyoxylate aminotransferase (AGT), a gene that has different functions in herbivores and carnivores, support this opinion. Additionally, there is evidence of positive selection for this gene among simian primates [78]. aBSREL detected evidence of episodic diversifying selection on two out of the 35 branches in the GBP5 phylogeny. A total of 35 branches underwent official testing to diversify their selection. The significance of the results was evaluated using the Likelihood Ratio Test at a significance level of p < 0.05 after adjusting for multiple comparisons (Fig 4). While episodic diversifying selection was found on 9 out of 29 branches in the GZMB phylogeny, aBSREL detected episodic diversifying selection on two branches out of a total of 35 branches in the IFNG phylogeny and 7 out of the 24 branches in the IRF7 phylogeny (Fig 4). aBSREL detected evidence of episodic diversifying selection on four out of 35 branches in the KLRD1 phylogeny and 8 out of the 29 branches in the RTP4 phylogeny (Fig 4).

Furthermore, the mammalian innate and adaptive immune systems are examples of evolutionary adaptations that were most likely motivated, at least in part, by the need to control the gut microbiota’s composition in ways that enhance host fitness [79]. The immune system offers mechanisms for distinguishing and eliminating harmful germs while allowing healthy or commensal microbes to coexist [69]. Studies have demonstrated that the body’s lack of immunological components might harm the gut microbiota composition in hosts. For instance, it has been demonstrated that removing Toll-like receptors from the host genome causes disruptions in the makeup of the gut microbiota in mice, which modifies the host’s energy harvesting and metabolism in likely maladaptive ways [80]. The GARD analysis detected recombination breakpoints in the GBP5, GZMB, IRF7, KLRD1, RTP4, and TRAT1 genes. GARD analyzed a total of 13,556 models at a speed of 21.42 models per second. The alignment consisted of 1183 possible breakpoints, resulting in a search space of 6358 models with a maximum of 7 breakpoints. However, the genetic algorithm only searched 0.00% of this search space (Fig 6). The AICc score of the best-fitting GARD model, which permits different topologies between segments (29983.2), is compared to that of the model assuming the same tree for all partitions inferred by GARD but allowing different branch lengths between partitions (30120.2). This suggests that the multiple-tree model may be preferred over the single-tree model by an evidence ratio of 100 or greater. This indicates that at least one of the breakpoints represents a genuine topological incongruence. GARD did not detect any signs of recombination in the IFNG and TNFSF4 genes. GARD analyzed a total of 2630 models at a speed of 49.62 models per second. The alignment consisted of 409 possible breakpoints, resulting in a search space of 409 models with a maximum of 1 breakpoint (Fig 6). The genetic algorithm examined 643.03% of this search space. The comparison of the AICc scores between the best-fitting GARD model, which permits different topologies between segments (9309.1), and the model that assumes the same tree for all partitions inferred by GARD but allows different branch lengths between partitions (9309.1), indicates that the multiple tree model cannot be favored over the single tree model by an evidence ratio of 100 or more. This suggests that some or all breakpoints may indicate rate variation rather than topological incongruence. Similarly, the makeup of the gut microbiota is different in RAG1^−/−^ mice, who do not have adaptive immune systems. While all mammalian species have gut microbiota, the evolutionary consequences of interacting with gut microbiota probably vary from mammalian to mammalian taxon [81]. According to recent investigations, mammalian orders exhibit varying degrees of concordance between the gut microbiota makeup and the host’s phylogenetic history [82]. While the gut microbiotas of certain animal orders like bats show relatively minor relationships with host phylogeny, the gut microbiotas of most other orders exhibit robust evidence of phylogenetic signal. Mammalian species may be spared the risk of evolutionary reliance on a particular gut microbiota if there is no species-specific gut microbiome [83]. In these circumstances, hosts might only evolve to incorporate signals from ambient or non-specific microorganisms into their growth, as opposed to signals from particular bacteria or microbes. On the other hand, hosts might stop depending on microbes for development [84]. These theories highlight the necessity for manipulation studies, including a greater variety of mammalian species with gut microbiotas that differ in phylogenetic signal. While this study has highlighted key interactions between gut microbiota and the adaptive immune system in various mammalian species, vast unexplored territory remains concerning how these interactions vary across ecological niches and evolutionary histories. Future research could focus on a broader array of species, particularly those from understudied environments, such as deep-sea mammals or high-altitude species. Investigating how extreme environmental pressures influence microbiota-immune system co-evolution could provide new insights into adaptive strategies. Another promising direction is the longitudinal study of immune system development from infancy to adulthood in various mammalian species. This would allow researchers to observe how the immune system evolves and adapts in response to environmental changes, infections, and diet across different life stages. Such studies could shed light on the timing and triggers of key immune system adaptations and how these might differ between species with varying life histories.

The specificity of the immune system and gut bacteria interactions represents a major knowledge gap. The entire range of microbial species and their distinct roles in immunological regulation are yet unknown, despite some, such as *Bacteroides fragilis* and segmented filamentous bacteria (SFB), have been identified as important participants. More thorough research is required to investigate the function of lesser-known microbial species and their interactions with various immune system components. Furthermore, it is yet unclear how particular microbial metabolites affect the development and functionality of immune cells.

## Conclusion

This study provides a comprehensive evolutionary and functional analysis of eight critical immune-related genes—GBP5, GZMB, IFNG, IRF7, KLRD1, RTP4, TNFSF4, and TRAT1—revealing their adaptive evolution across mammalian species. Our findings demonstrate that these genes have undergone significant positive selection, particularly in functional domains essential for pathogen recognition and immune regulation. The high correlation in their phylogenetic trees (Pearson r = 0.84–0.95) supports their co-evolution, likely driven by shared selective pressures from pathogens. The identification of positively selected sites within conserved domains, such as GBP5’s GTPase region and GZMB’s serine protease active site, suggests that these genes have fine-tuned their functions to enhance host defense while maintaining core immune activities. A key discovery was the variability in recombination rates among these genes. While GBP5, GZMB, and IRF7 exhibited significant recombination breakpoints—potentially facilitating rapid adaptation—IFNG and TNFSF4 remained stable, possibly due to stronger functional constraints. This dichotomy highlights the balance between evolutionary flexibility and conserved immune mechanisms. The branch-site selection analysis further revealed episodic diversifying selection, with 15–26 branches under significant positive selection, indicating that these genes have repeatedly adapted to emerging pathogenic threats throughout mammalian evolution. Functional annotation linked the selected sites to critical immune processes, including inflammasome activation (GBP5), cytotoxic T-cell function (GZMB), and interferon-mediated antiviral responses (IFNG, IRF7). Tissue-specific expression analysis reinforced their roles in immune-active tissues, with notable enrichment in whole blood, spleen, and lymphocytes. Pathway analysis further connected these genes to Th1/Th2 differentiation and cytokine regulation, underscoring their importance in adaptive immunity. These findings have important implications for biomedical research. The positively selected sites identified in this study may serve as targets for immunotherapy or vaccine development, particularly in diseases where immune evasion is a challenge. Additionally, the observed co-evolutionary patterns suggest that these genes function as an integrated network, warranting further investigation into their synergistic roles in host defense.

## Supporting information

S1 Table Mapping of Positively Selected Sites to Functional Protein Domains. This table maps the amino acid sites identified under positive selection (Posterior Probability > 0.95 from PAML BEB analysis) to known functional domains and discusses their potential functional implications in the context of host-pathogen co-evolution.
(DOCX)

S2 Table Data Source Accession Numbers. This table provides the NCBI Gene ID and Ensembl Gene ID for the eight immune-related genes analyzed across the 42 mammalian species in this study. These accession numbers serve as unique identifiers for the gene sequences retrieved from the NCBI and Ensembl databases. Blank cells indicate that a standard, curated gene record for that species was not available in the respective database at the time of data collection and an alternative genomic scaffold was used for analysis.
(DOCX)
