# Expression of Concern: Structural and evolutionary constraints shape adaptive landscapes of immune-related genes across mammalian phylogeny

**DOI:** 10.1371/journal.pone.0355444

**Published:** 2026-08-06

**Authors:** The PLOS One Editors

After this article [1] was published, the *PLOS One* Editors noted concerns about the article’s peer review and compliance with the PLOS Authorship policy.

In light of the cumulative issues, the *PLOS One* Editors issue this Expression of Concern. Readers are advised to interpret the article [1] with caution.
