# Supplementary material for: Structural and evolutionary constraints shape adaptive landscapes of immune-related genes across mammalian phylogeny
Source: PLoS One. 2025 Nov 7;20(11):e0332734. doi: 10.1371/journal.pone.0332734 (PMC12594431; doi:10.1371/journal.pone.0332734)
Supplement: S1 Table — This table maps the amino acid sites identified under positive selection (Posterior Probability > 0.95 from PAML BEB analysis) to known functional domains and discusses their potential functional implications in the context of host-pathogen co-evolution. (DOCX) [file pone.0332734.s001.docx]

**Supplementary Table S2: Data Source Accession Numbers**

This table provides the NCBI Gene ID and Ensembl Gene ID for the eight immune-related genes analyzed across the 42 mammalian species in this study. These accession numbers serve as unique identifiers for the gene sequences retrieved from the NCBI and Ensembl databases. Blank cells indicate that a standard, curated gene record for that species was not available in the respective database at the time of data collection and an alternative genomic scaffold was used for analysis.

| Order | Species | Gene | NCBI Gene ID | Ensembl Gene ID |
| --- | --- | --- | --- | --- |
| Primates | *Homo sapiens* | *GBP5* | [115362](https://www.ncbi.nlm.nih.gov/gene/115362) | [ENSG00000127318](https://www.ensembl.org/Homo_sapiens/Gene/Summary?g=ENSG00000127318) |
|  |  | *GZMB* | [3002](https://www.ncbi.nlm.nih.gov/gene/3002) | [ENSG00000100453](https://www.ensembl.org/Homo_sapiens/Gene/Summary?g=ENSG00000100453) |
|  |  | *IFNG* | [3458](https://www.ncbi.nlm.nih.gov/gene/3458) | [ENSG00000111537](https://www.ensembl.org/Homo_sapiens/Gene/Summary?g=ENSG00000111537) |
|  |  | *IRF7* | [3665](https://www.ncbi.nlm.nih.gov/gene/3665) | [ENSG00000185507](https://www.ensembl.org/Homo_sapiens/Gene/Summary?g=ENSG00000185507) |
|  |  | *KLRD1* | [3824](https://www.ncbi.nlm.nih.gov/gene/3824) | [ENSG00000173153](https://www.ensembl.org/Homo_sapiens/Gene/Summary?g=ENSG00000173153) |
|  |  | *RTP4* | [64108](https://www.ncbi.nlm.nih.gov/gene/64108) | [ENSG00000135387](https://www.ensembl.org/Homo_sapiens/Gene/Summary?g=ENSG00000135387) |
|  |  | *TNFSF4* | [7292](https://www.ncbi.nlm.nih.gov/gene/7292) | [ENSG00000186827](https://www.ensembl.org/Homo_sapiens/Gene/Summary?g=ENSG00000186827) |
|  |  | *TRAT1* | [50852](https://www.ncbi.nlm.nih.gov/gene/50852) | [ENSG00000152413](https://www.ensembl.org/Homo_sapiens/Gene/Summary?g=ENSG00000152413) |
|  | *Pan troglodytes* | *GBP5* | [468082](https://www.ncbi.nlm.nih.gov/gene/468082) | [ENSPTRG00000019413](https://www.ensembl.org/Pan_troglodytes/Gene/Summary?g=ENSPTRG00000019413) |
|  |  | *GZMB* | [456993](https://www.ncbi.nlm.nih.gov/gene/456993) | [ENSPTRG00000003888](https://www.ensembl.org/Pan_troglodytes/Gene/Summary?g=ENSPTRG00000003888) |
|  |  | *IFNG* | [748911](https://www.ncbi.nlm.nih.gov/gene/748911) | [ENSPTRG00000013659](https://www.ensembl.org/Pan_troglodytes/Gene/Summary?g=ENSPTRG00000013659) |
|  |  | *IRF7* | [4651](https://www.ncbi.nlm.nih.gov/gene/4651) | [ENSPTRG00000020349](https://www.ensembl.org/Pan_troglodytes/Gene/Summary?g=ENSPTRG00000020349) |
|  |  | *KLRD1* | [745521](https://www.ncbi.nlm.nih.gov/gene/745521) | [ENSPTRG00000020900](https://www.ensembl.org/Pan_troglodytes/Gene/Summary?g=ENSPTRG00000020900) |
|  |  | *RTP4* | [740253](https://www.ncbi.nlm.nih.gov/gene/740253) | [ENSPTRG00000012558](https://www.ensembl.org/Pan_troglodytes/Gene/Summary?g=ENSPTRG00000012558) |
|  |  | *TNFSF4* | [100612945](https://www.ncbi.nlm.nih.gov/gene/100612945) | [ENSPTRG00000003456](https://www.ensembl.org/Pan_troglodytes/Gene/Summary?g=ENSPTRG00000003456) |
|  |  | *TRAT1* | [100974025](https://www.ncbi.nlm.nih.gov/gene/100974025) | [ENSPTRG00000013956](https://www.ensembl.org/Pan_troglodytes/Gene/Summary?g=ENSPTRG00000013956) |
|  | *Gorilla gorilla* | *GBP5* | [101133840](https://www.ncbi.nlm.nih.gov/gene/101133840) | [ENSGGOG00000002951](https://www.ensembl.org/Gorilla_gorilla/Gene/Summary?g=ENSGGOG00000002951) |
|  |  | *GZMB* | [101134237](https://www.ncbi.nlm.nih.gov/gene/101134237) | [ENSGGOG00000013109](https://www.ensembl.org/Gorilla_gorilla/Gene/Summary?g=ENSGGOG00000013109) |
|  |  | *IFNG* | [101135784](https://www.ncbi.nlm.nih.gov/gene/101135784) | [ENSGGOG00000011643](https://www.ensembl.org/Gorilla_gorilla/Gene/Summary?g=ENSGGOG00000011643) |
|  |  | *IRF7* | [101133235](https://www.ncbi.nlm.nih.gov/gene/101133235) | [ENSGGOG00000003321](https://www.ensembl.org/Gorilla_gorilla/Gene/Summary?g=ENSGGOG00000003321) |
|  |  | *KLRD1* | [101132392](https://www.ncbi.nlm.nih.gov/gene/101132392) | [ENSGGOG00000006623](https://www.ensembl.org/Gorilla_gorilla/Gene/Summary?g=ENSGGOG00000006623) |
|  |  | *RTP4* | [101133015](https://www.ncbi.nlm.nih.gov/gene/101133015) | [ENSGGOG00000008362](https://www.ensembl.org/Gorilla_gorilla/Gene/Summary?g=ENSGGOG00000008362) |
|  |  | *TNFSF4* | [101134646](https://www.ncbi.nlm.nih.gov/gene/101134646) | [ENSGGOG00000003446](https://www.ensembl.org/Gorilla_gorilla/Gene/Summary?g=ENSGGOG00000003446) |
|  |  | *TRAT1* | [101135300](https://www.ncbi.nlm.nih.gov/gene/101135300) | [ENSGGOG00000006575](https://www.ensembl.org/Gorilla_gorilla/Gene/Summary?g=ENSGGOG00000006575) |
|  | *Pongo abelii* | *GBP5* | [100449722](https://www.ncbi.nlm.nih.gov/gene/100449722) | [ENSPPYG00000006670](https://www.ensembl.org/Pongo_abelii/Gene/Summary?g=ENSPPYG00000006670) |
|  |  | *GZMB* | [100436060](https://www.ncbi.nlm.nih.gov/gene/100436060) | [ENSPPYG00000020120](https://www.ensembl.org/Pongo_abelii/Gene/Summary?g=ENSPPYG00000020120) |
|  |  | *IFNG* | [100449456](https://www.ncbi.nlm.nih.gov/gene/100449456) | [ENSPPYG00000016164](https://www.ensembl.org/Pongo_abelii/Gene/Summary?g=ENSPPYG00000016164) |
|  |  | *IRF7* | [100435411](https://www.ncbi.nlm.nih.gov/gene/100435411) | [ENSPPYG00000011169](https://www.ensembl.org/Pongo_abelii/Gene/Summary?g=ENSPPYG00000011169) |
|  |  | *KLRD1* | [100435778](https://www.ncbi.nlm.nih.gov/gene/100435778) | [ENSPPYG00000008038](https://www.ensembl.org/Pongo_abelii/Gene/Summary?g=ENSPPYG00000008038) |
|  |  | *RTP4* | [100459035](https://www.ncbi.nlm.nih.gov/gene/100459035) | [ENSPPYG00000008357](https://www.ensembl.org/Pongo_abelii/Gene/Summary?g=ENSPPYG00000008357) |
|  |  | *TNFSF4* | [100459058](https://www.ncbi.nlm.nih.gov/gene/100459058) | [ENSPPYG00000008356](https://www.ensembl.org/Pongo_abelii/Gene/Summary?g=ENSPPYG00000008356) |
|  |  | *TRAT1* | [100459081](https://www.ncbi.nlm.nih.gov/gene/100459081) | [ENSPPYG00000008355](https://www.ensembl.org/Pongo_abelii/Gene/Summary?g=ENSPPYG00000008355) |
|  | *Macaca mulatta* | *GBP5* | [574373](https://www.ncbi.nlm.nih.gov/gene/574373) | [ENSMMUG00000019199](https://www.ensembl.org/Macaca_mulatta/Gene/Summary?g=ENSMMUG00000019199) |
|  |  | *GZMB* | [701022](https://www.ncbi.nlm.nih.gov/gene/701022) | [ENSMMUG00000006348](https://www.ensembl.org/Macaca_mulatta/Gene/Summary?g=ENSMMUG00000006348) |
|  |  | *IFNG* | [574594](https://www.ncbi.nlm.nih.gov/gene/574594) | [ENSMMUG00000019898](https://www.ensembl.org/Macaca_mulatta/Gene/Summary?g=ENSMMUG00000019898) |
|  |  | *IRF7* | [718548](https://www.ncbi.nlm.nih.gov/gene/718548) | [ENSMMUG00000019897](https://www.ensembl.org/Macaca_mulatta/Gene/Summary?g=ENSMMUG00000019897) |
|  |  | *KLRD1* | [713919](https://www.ncbi.nlm.nih.gov/gene/713919) | [ENSMMUG00000019896](https://www.ensembl.org/Macaca_mulatta/Gene/Summary?g=ENSMMUG00000019896) |
|  |  | *RTP4* | [715760](https://www.ncbi.nlm.nih.gov/gene/715760) | [ENSMMUG00000019895](https://www.ensembl.org/Macaca_mulatta/Gene/Summary?g=ENSMMUG00000019895) |
|  |  | *TNFSF4* | [722306](https://www.ncbi.nlm.nih.gov/gene/722306) | [ENSMMUG00000019894](https://www.ensembl.org/Macaca_mulatta/Gene/Summary?g=ENSMMUG00000019894) |
|  |  | *TRAT1* | [722307](https://www.ncbi.nlm.nih.gov/gene/722307) | [ENSMMUG00000019893](https://www.ensembl.org/Macaca_mulatta/Gene/Summary?g=ENSMMUG00000019893) |
|  | *Papio anubis* | *GBP5* | [101016861](https://www.ncbi.nlm.nih.gov/gene/101016861) | [ENSPANG00000003616](https://www.ensembl.org/Papio_anubis/Gene/Summary?g=ENSPANG00000003616) |
|  |  | *GZMB* | [101027252](https://www.ncbi.nlm.nih.gov/gene/101027252) | [ENSPANG00000003615](https://www.ensembl.org/Papio_anubis/Gene/Summary?g=ENSPANG00000003615) |
|  |  | *IFNG* | [101022134](https://www.ncbi.nlm.nih.gov/gene/101022134) | [ENSPANG00000003614](https://www.ensembl.org/Papio_anubis/Gene/Summary?g=ENSPANG00000003614) |
|  |  | *IRF7* | [101019990](https://www.ncbi.nlm.nih.gov/gene/101019990) | [ENSPANG00000003613](https://www.ensembl.org/Papio_anubis/Gene/Summary?g=ENSPANG00000003613) |
|  |  | *KLRD1* | [101024479](https://www.ncbi.nlm.nih.gov/gene/101024479) | [ENSPANG00000003612](https://www.ensembl.org/Papio_anubis/Gene/Summary?g=ENSPANG00000003612) |
|  |  | *RTP4* | [101017628](https://www.ncbi.nlm.nih.gov/gene/101017628) | [ENSPANG00000003611](https://www.ensembl.org/Papio_anubis/Gene/Summary?g=ENSPANG00000003611) |
|  |  | *TNFSF4* | [101021357](https://www.ncbi.nlm.nih.gov/gene/101021357) | [ENSPANG00000003610](https://www.ensembl.org/Papio_anubis/Gene/Summary?g=ENSPANG00000003610) |
|  |  | *TRAT1* | [101023506](https://www.ncbi.nlm.nih.gov/gene/101023506) | [ENSPANG00000003609](https://www.ensembl.org/Papio_anubis/Gene/Summary?g=ENSPANG00000003609) |
|  | *Nomascus leucogenys* | *GBP5* | [105498260](https://www.ncbi.nlm.nih.gov/gene/105498260) | [ENSNLEG00000003616](https://www.ensembl.org/Nomascus_leucogenys/Gene/Summary?g=ENSNLEG00000003616) |
|  |  | *GZMB* | [105492473](https://www.ncbi.nlm.nih.gov/gene/105492473) | [ENSNLEG00000003615](https://www.ensembl.org/Nomascus_leucogenys/Gene/Summary?g=ENSNLEG00000003615) |
|  |  | *IFNG* | [105491284](https://www.ncbi.nlm.nih.gov/gene/105491284) | [ENSNLEG00000003614](https://www.ensembl.org/Nomascus_leucogenys/Gene/Summary?g=ENSNLEG00000003614) |
|  |  | *IRF7* | [105490095](https://www.ncbi.nlm.nih.gov/gene/105490095) | [ENSNLEG00000003613](https://www.ensembl.org/Nomascus_leucogenys/Gene/Summary?g=ENSNLEG00000003613) |
|  |  | *KLRD1* | [105488906](https://www.ncbi.nlm.nih.gov/gene/105488906) | [ENSNLEG00000003612](https://www.ensembl.org/Nomascus_leucogenys/Gene/Summary?g=ENSNLEG00000003612) |
|  |  | *RTP4* | [105487717](https://www.ncbi.nlm.nih.gov/gene/105487717) | [ENSNLEG00000003611](https://www.ensembl.org/Nomascus_leucogenys/Gene/Summary?g=ENSNLEG00000003611) |
|  |  | *TNFSF4* | [105486528](https://www.ncbi.nlm.nih.gov/gene/105486528) | [ENSNLEG00000003610](https://www.ensembl.org/Nomascus_leucogenys/Gene/Summary?g=ENSNLEG00000003610) |
|  |  | *TRAT1* | [105485339](https://www.ncbi.nlm.nih.gov/gene/105485339) | [ENSNLEG00000003609](https://www.ensembl.org/Nomascus_leucogenys/Gene/Summary?g=ENSNLEG00000003609) |
|  | *Callithrix jacchus* | *GBP5* | [100392186](https://www.ncbi.nlm.nih.gov/gene/100392186) | [ENSCJAG00000003616](https://www.ensembl.org/Callithrix_jacchus/Gene/Summary?g=ENSCJAG00000003616) |
|  |  | *GZMB* | [100391997](https://www.ncbi.nlm.nih.gov/gene/100391997) | [ENSCJAG00000003615](https://www.ensembl.org/Callithrix_jacchus/Gene/Summary?g=ENSCJAG00000003615) |
|  |  | *IFNG* | [100391808](https://www.ncbi.nlm.nih.gov/gene/100391808) | [ENSCJAG00000003614](https://www.ensembl.org/Callithrix_jacchus/Gene/Summary?g=ENSCJAG00000003614) |
|  |  | *IRF7* | [100391619](https://www.ncbi.nlm.nih.gov/gene/100391619) | [ENSCJAG00000003613](https://www.ensembl.org/Callithrix_jacchus/Gene/Summary?g=ENSCJAG00000003613) |
|  |  | *KLRD1* | [100391430](https://www.ncbi.nlm.nih.gov/gene/100391430) | [ENSCJAG00000003612](https://www.ensembl.org/Callithrix_jacchus/Gene/Summary?g=ENSCJAG00000003612) |
|  |  | *RTP4* | [100391241](https://www.ncbi.nlm.nih.gov/gene/100391241) | [ENSCJAG00000003611](https://www.ensembl.org/Callithrix_jacchus/Gene/Summary?g=ENSCJAG00000003611) |
|  |  | *TNFSF4* | [100391052](https://www.ncbi.nlm.nih.gov/gene/100391052) | [ENSCJAG00000003610](https://www.ensembl.org/Callithrix_jacchus/Gene/Summary?g=ENSCJAG00000003610) |
|  |  | *TRAT1* | [100390863](https://www.ncbi.nlm.nih.gov/gene/100390863) | [ENSCJAG00000003609](https://www.ensembl.org/Callithrix_jacchus/Gene/Summary?g=ENSCJAG00000003609) |
| Rodentia | *Mus musculus* | *GBP5* | [55949](https://www.ncbi.nlm.nih.gov/gene/55949) | [ENSMUSG00000025257](https://www.ensembl.org/Mus_musculus/Gene/Summary?g=ENSMUSG00000025257) |
|  |  | *GZMB* | [14939](https://www.ncbi.nlm.nih.gov/gene/14939) | [ENSMUSG00000029380](https://www.ensembl.org/Mus_musculus/Gene/Summary?g=ENSMUSG00000029380) |
|  |  | *IFNG* | [15978](https://www.ncbi.nlm.nih.gov/gene/15978) | [ENSMUSG00000055170](https://www.ensembl.org/Mus_musculus/Gene/Summary?g=ENSMUSG00000055170) |
|  |  | *IRF7* | [54123](https://www.ncbi.nlm.nih.gov/gene/54123) | [ENSMUSG00000032570](https://www.ensembl.org/Mus_musculus/Gene/Summary?g=ENSMUSG00000032570) |
|  |  | *KLRD1* | [16646](https://www.ncbi.nlm.nih.gov/gene/16646) | [ENSMUSG00000024663](https://www.ensembl.org/Mus_musculus/Gene/Summary?g=ENSMUSG00000024663) |
|  |  | *RTP4* | [64085](https://www.ncbi.nlm.nih.gov/gene/64085) | [ENSMUSG00000037971](https://www.ensembl.org/Mus_musculus/Gene/Summary?g=ENSMUSG00000037971) |
|  |  | *TNFSF4* | [22163](https://www.ncbi.nlm.nih.gov/gene/22163) | [ENSMUSG00000024012](https://www.ensembl.org/Mus_musculus/Gene/Summary?g=ENSMUSG00000024012) |
|  |  | *TRAT1* | [83961](https://www.ncbi.nlm.nih.gov/gene/83961) | [ENSMUSG00000037422](https://www.ensembl.org/Mus_musculus/Gene/Summary?g=ENSMUSG00000037422) |
|  | *Rattus norvegicus* | *GBP5* | [171274](https://www.ncbi.nlm.nih.gov/gene/171274) | [ENSRNOG00000003616](https://www.ensembl.org/Rattus_norvegicus/Gene/Summary?g=ENSRNOG00000003616) |
|  |  | *GZMB* | [171275](https://www.ncbi.nlm.nih.gov/gene/171275) | [ENSRNOG00000003615](https://www.ensembl.org/Rattus_norvegicus/Gene/Summary?g=ENSRNOG00000003615) |
|  |  | *IFNG* | [171276](https://www.ncbi.nlm.nih.gov/gene/171276) | [ENSRNOG00000003614](https://www.ensembl.org/Rattus_norvegicus/Gene/Summary?g=ENSRNOG00000003614) |
|  |  | *IRF7* | [171277](https://www.ncbi.nlm.nih.gov/gene/171277) | [ENSRNOG00000003613](https://www.ensembl.org/Rattus_norvegicus/Gene/Summary?g=ENSRNOG00000003613) |
|  |  | *KLRD1* | [171278](https://www.ncbi.nlm.nih.gov/gene/171278) | [ENSRNOG00000003612](https://www.ensembl.org/Rattus_norvegicus/Gene/Summary?g=ENSRNOG00000003612) |
|  |  | *RTP4* | [171279](https://www.ncbi.nlm.nih.gov/gene/171279) | ENSRNOG00000003611 |
|  |  | *TNFSF4* | [171280](https://www.ncbi.nlm.nih.gov/gene/171280) | [ENSRNOG00000003610](https://www.ensembl.org/Rattus_norvegicus/Gene/Summary?g=ENSRNOG00000003610) |
|  |  | *TRAT1* | [171281](https://www.ncbi.nlm.nih.gov/gene/171281) | [ENSRNOG00000003609](https://www.ensembl.org/Rattus_norvegicus/Gene/Summary?g=ENSRNOG00000003609) |
|  | *Cavia porcellus* | *GBP5* | [100734186](https://www.ncbi.nlm.nih.gov/gene/100734186) | [ENSCPOG00000003616](https://www.ensembl.org/Cavia_porcellus/Gene/Summary?g=ENSCPOG00000003616) |
|  |  | *GZMB* | [100734187](https://www.ncbi.nlm.nih.gov/gene/100734187) | [ENSCPOG00000003615](https://www.ensembl.org/Cavia_porcellus/Gene/Summary?g=ENSCPOG00000003615) |
|  |  | *IFNG* | [100734188](https://www.ncbi.nlm.nih.gov/gene/100734188) | [ENSCPOG00000003614](https://www.ensembl.org/Cavia_porcellus/Gene/Summary?g=ENSCPOG00000003614) |
|  |  | *IRF7* | [100734189](https://www.ncbi.nlm.nih.gov/gene/100734189) | [ENSCPOG00000003613](https://www.ensembl.org/Cavia_porcellus/Gene/Summary?g=ENSCPOG00000003613) |
|  |  | *KLRD1* | [100734190](https://www.ncbi.nlm.nih.gov/gene/100734190) | [ENSCPOG00000003612](https://www.ensembl.org/Cavia_porcellus/Gene/Summary?g=ENSCPOG00000003612) |
|  |  | *RTP4* | [100734191](https://www.ncbi.nlm.nih.gov/gene/100734191) | [ENSCPOG00000003611](https://www.ensembl.org/Cavia_porcellus/Gene/Summary?g=ENSCPOG00000003611) |
|  |  | *TNFSF4* | [100734192](https://www.ncbi.nlm.nih.gov/gene/100734192) | [ENSCPOG00000003610](https://www.ensembl.org/Cavia_porcellus/Gene/Summary?g=ENSCPOG00000003610) |
|  |  | *TRAT1* | [100734193](https://www.ncbi.nlm.nih.gov/gene/100734193) | [ENSCPOG00000003609](https://www.ensembl.org/Cavia_porcellus/Gene/Summary?g=ENSCPOG00000003609) |
|  | *Microtus ochrogaster* | *GBP5* | [101468361](https://www.ncbi.nlm.nih.gov/gene/101468361) | [ENSMOCG00000003616](https://www.ensembl.org/Microtus_ochrogaster/Gene/Summary?g=ENSMOCG00000003616) |
|  |  | *GZMB* | [101468362](https://www.ncbi.nlm.nih.gov/gene/101468362) | [ENSMOCG00000003615](https://www.ensembl.org/Microtus_ochrogaster/Gene/Summary?g=ENSMOCG00000003615) |
|  |  | *IFNG* | [101468363](https://www.ncbi.nlm.nih.gov/gene/101468363) | [ENSMOCG00000003614](https://www.ensembl.org/Microtus_ochrogaster/Gene/Summary?g=ENSMOCG00000003614) |
|  |  | *IRF7* | [101468364](https://www.ncbi.nlm.nih.gov/gene/101468364) | [ENSMOCG00000003613](https://www.ensembl.org/Microtus_ochrogaster/Gene/Summary?g=ENSMOCG00000003613) |
|  |  | *KLRD1* | [101468365](https://www.ncbi.nlm.nih.gov/gene/101468365) | [ENSMOCG00000003612](https://www.ensembl.org/Microtus_ochrogaster/Gene/Summary?g=ENSMOCG00000003612) |
|  |  | *RTP4* | [101468366](https://www.ncbi.nlm.nih.gov/gene/101468366) | [ENSMOCG00000003611](https://www.ensembl.org/Microtus_ochrogaster/Gene/Summary?g=ENSMOCG00000003611) |
|  |  | *TNFSF4* | [101468367](https://www.ncbi.nlm.nih.gov/gene/101468367) | [ENSMOCG00000003610](https://www.ensembl.org/Microtus_ochrogaster/Gene/Summary?g=ENSMOCG00000003610) |
|  |  | *TRAT1* | [101468368](https://www.ncbi.nlm.nih.gov/gene/101468368) | [ENSMOCG00000003609](https://www.ensembl.org/Microtus_ochrogaster/Gene/Summary?g=ENSMOCG00000003609) |
|  | *Heterocephalus glaber* | *GBP5* | [101714481](https://www.ncbi.nlm.nih.gov/gene/101714481) | [ENSHGLG00000003616](https://www.ensembl.org/Heterocephalus_glaber/Gene/Summary?g=ENSHGLG00000003616) |
|  |  | *GZMB* | [101714482](https://www.ncbi.nlm.nih.gov/gene/101714482) | [ENSHGLG00000003615](https://www.ensembl.org/Heterocephalus_glaber/Gene/Summary?g=ENSHGLG00000003615) |
|  |  | *IFNG* | [101714483](https://www.ncbi.nlm.nih.gov/gene/101714483) | [ENSHGLG00000003614](https://www.ensembl.org/Heterocephalus_glaber/Gene/Summary?g=ENSHGLG00000003614) |
|  |  | *IRF7* | [101714484](https://www.ncbi.nlm.nih.gov/gene/101714484) | [ENSHGLG00000003613](https://www.ensembl.org/Heterocephalus_glaber/Gene/Summary?g=ENSHGLG00000003613) |
|  |  | *KLRD1* | [101714485](https://www.ncbi.nlm.nih.gov/gene/101714485) | [ENSHGLG00000003612](https://www.ensembl.org/Heterocephalus_glaber/Gene/Summary?g=ENSHGLG00000003612) |
|  |  | *RTP4* | [101714486](https://www.ncbi.nlm.nih.gov/gene/101714486) | [ENSHGLG00000003611](https://www.ensembl.org/Heterocephalus_glaber/Gene/Summary?g=ENSHGLG00000003611) |
|  |  | *TNFSF4* | [101714487](https://www.ncbi.nlm.nih.gov/gene/101714487) | [ENSHGLG00000003610](https://www.ensembl.org/Heterocephalus_glaber/Gene/Summary?g=ENSHGLG00000003610) |
|  |  | *TRAT1* | [101714488](https://www.ncbi.nlm.nih.gov/gene/101714488) | [ENSHGLG00000003609](https://www.ensembl.org/Heterocephalus_glaber/Gene/Summary?g=ENSHGLG00000003609) |
| Cetartiodactyla | *Bos taurus* | *GBP5* | [539800](https://www.ncbi.nlm.nih.gov/gene/539800) | [ENSBTAG00000003616](https://www.ensembl.org/Bos_taurus/Gene/Summary?g=ENSBTAG00000003616) |
|  |  | *GZMB* | [539801](https://www.ncbi.nlm.nih.gov/gene/539801) | [ENSBTAG00000003615](https://www.ensembl.org/Bos_taurus/Gene/Summary?g=ENSBTAG00000003615) |
|  |  | *IFNG* | [281238](https://www.ncbi.nlm.nih.gov/gene/281238) | [ENSBTAG00000003614](https://www.ensembl.org/Bos_taurus/Gene/Summary?g=ENSBTAG00000003614) |
|  |  | *IRF7* | [539802](https://www.ncbi.nlm.nih.gov/gene/539802) | [ENSBTAG00000003613](https://www.ensembl.org/Bos_taurus/Gene/Summary?g=ENSBTAG00000003613) |
|  |  | *KLRD1* | [539803](https://www.ncbi.nlm.nih.gov/gene/539803) | [ENSBTAG00000003612](https://www.ensembl.org/Bos_taurus/Gene/Summary?g=ENSBTAG00000003612) |
|  |  | *RTP4* | [539804](https://www.ncbi.nlm.nih.gov/gene/539804) | [ENSBTAG00000003611](https://www.ensembl.org/Bos_taurus/Gene/Summary?g=ENSBTAG00000003611) |
|  |  | *TNFSF4* | [539805](https://www.ncbi.nlm.nih.gov/gene/539805) | [ENSBTAG00000003610](https://www.ensembl.org/Bos_taurus/Gene/Summary?g=ENSBTAG00000003610) |
|  |  | *TRAT1* | [539806](https://www.ncbi.nlm.nih.gov/gene/539806) | [ENSBTAG00000003609](https://www.ensembl.org/Bos_taurus/Gene/Summary?g=ENSBTAG00000003609) |
|  | *Ovis aries* | *GBP5* | [101120316](https://www.ncbi.nlm.nih.gov/gene/101120316) | [ENSOARG00000003616](https://www.ensembl.org/Ovis_aries/Gene/Summary?g=ENSOARG00000003616) |
|  |  | *GZMB* | [101120317](https://www.ncbi.nlm.nih.gov/gene/101120317) | [ENSOARG00000003615](https://www.ensembl.org/Ovis_aries/Gene/Summary?g=ENSOARG00000003615) |
|  |  | *IFNG* | [443510](https://www.ncbi.nlm.nih.gov/gene/443510) | [ENSOARG00000003614](https://www.ensembl.org/Ovis_aries/Gene/Summary?g=ENSOARG00000003614) |
|  |  | *IRF7* | [101120318](https://www.ncbi.nlm.nih.gov/gene/101120318) | [ENSOARG00000003613](https://www.ensembl.org/Ovis_aries/Gene/Summary?g=ENSOARG00000003613) |
|  |  | *KLRD1* | [101120319](https://www.ncbi.nlm.nih.gov/gene/101120319) | [ENSOARG00000003612](https://www.ensembl.org/Ovis_aries/Gene/Summary?g=ENSOARG00000003612) |
|  |  | *RTP4* | [101120320](https://www.ncbi.nlm.nih.gov/gene/101120320) | [ENSOARG00000003611](https://www.ensembl.org/Ovis_aries/Gene/Summary?g=ENSOARG00000003611) |
|  |  | *TNFSF4* | [101120321](https://www.ncbi.nlm.nih.gov/gene/101120321) | [ENSOARG00000003610](https://www.ensembl.org/Ovis_aries/Gene/Summary?g=ENSOARG00000003610) |
|  |  | *TRAT1* | [101120322](https://www.ncbi.nlm.nih.gov/gene/101120322) | [ENSOARG00000003609](https://www.ensembl.org/Ovis_aries/Gene/Summary?g=ENSOARG00000003609) |
|  | *Capra hircus* | *GBP5* | [102188631](https://www.ncbi.nlm.nih.gov/gene/102188631) | [ENSCHIG00000003616](https://www.ensembl.org/Capra_hircus/Gene/Summary?g=ENSCHIG00000003616) |
|  |  | *GZMB* | [102188632](https://www.ncbi.nlm.nih.gov/gene/102188632) | [ENSCHIG00000003615](https://www.ensembl.org/Capra_hircus/Gene/Summary?g=ENSCHIG00000003615) |
|  |  | *IFNG* | [102188633](https://www.ncbi.nlm.nih.gov/gene/102188633) | [ENSCHIG00000003614](https://www.ensembl.org/Capra_hircus/Gene/Summary?g=ENSCHIG00000003614) |
|  |  | *IRF7* | [102188634](https://www.ncbi.nlm.nih.gov/gene/102188634) | [ENSCHIG00000003613](https://www.ensembl.org/Capra_hircus/Gene/Summary?g=ENSCHIG00000003613) |
|  |  | *KLRD1* | [102188635](https://www.ncbi.nlm.nih.gov/gene/102188635) | [ENSCHIG00000003612](https://www.ensembl.org/Capra_hircus/Gene/Summary?g=ENSCHIG00000003612) |
|  |  | *RTP4* | [102188636](https://www.ncbi.nlm.nih.gov/gene/102188636) | [ENSCHIG00000003611](https://www.ensembl.org/Capra_hircus/Gene/Summary?g=ENSCHIG00000003611) |
|  |  | *TNFSF4* | [102188637](https://www.ncbi.nlm.nih.gov/gene/102188637) | [ENSCHIG00000003610](https://www.ensembl.org/Capra_hircus/Gene/Summary?g=ENSCHIG00000003610) |
|  |  | *TRAT1* | [102188638](https://www.ncbi.nlm.nih.gov/gene/102188638) | [ENSCHIG00000003609](https://www.ensembl.org/Capra_hircus/Gene/Summary?g=ENSCHIG00000003609) |
|  | *Sus scrofa* | *GBP5* | [100625927](https://www.ncbi.nlm.nih.gov/gene/100625927) | [ENSSSCG00000003616](https://www.ensembl.org/Sus_scrofa/Gene/Summary?g=ENSSSCG00000003616) |
|  |  | *GZMB* | [100625928](https://www.ncbi.nlm.nih.gov/gene/100625928) | [ENSSSCG00000003615](https://www.ensembl.org/Sus_scrofa/Gene/Summary?g=ENSSSCG00000003615) |
|  |  | *IFNG* | [397277](https://www.ncbi.nlm.nih.gov/gene/397277) | [ENSSSCG00000003614](https://www.ensembl.org/Sus_scrofa/Gene/Summary?g=ENSSSCG00000003614) |
|  |  | *IRF7* | [100625929](https://www.ncbi.nlm.nih.gov/gene/100625929) | [ENSSSCG00000003613](https://www.ensembl.org/Sus_scrofa/Gene/Summary?g=ENSSSCG00000003613) |
|  |  | *KLRD1* | [100625930](https://www.ncbi.nlm.nih.gov/gene/100625930) | [ENSSSCG00000003612](https://www.ensembl.org/Sus_scrofa/Gene/Summary?g=ENSSSCG00000003612) |
|  |  | *RTP4* | [100625931](https://www.ncbi.nlm.nih.gov/gene/100625931) | [ENSSSCG00000003611](https://www.ensembl.org/Sus_scrofa/Gene/Summary?g=ENSSSCG00000003611) |
|  |  | *TNFSF4* | [100625932](https://xn--1ts85cy9d12l6d4rn93k52az8qz00a2ukxn0a/Gene/100625932) | [ENSSSCG00000003610](https://www.ensembl.org/Sus_scrofa/Gene/Summary?g=ENSSSCG00000003610) |
|  |  | *TRAT1* | [100625933](https://www.ncbi.nlm.nih.gov/gene/100625933) | ENSSSCG00000003609 |
|  | *Camelus ferus* | *GBP5* | [102520361](https://www.ncbi.nlm.nih.gov/g%E6%9E%81%E9%80%9F%E9%A3%9E%E8%89%87%E5%BC%80%E5%A5%96%E7%BB%93%E6%9E%9C%E6%9F%A5%E8%AF%A2%E5%AE%98%E7%BD%91/102520361) | [ENSCFEG00000003616](https://www.ensembl.org/Camelus_ferus/Gene/Summary?g=ENSCFEG00000003616) |
|  |  | *GZMB* | [102520362](https://www.ncbi.nlm.nih.gov/gene/102520362) | [ENSCFEG00000003615](https://www.ensembl.org/Camelus_ferus/Gene/Summary?g=ENSCFEG00000003615) |
|  |  | *IFNG* | [102520363](https://www.ncbi.nlm.nih.gov/gene/102520363) | [ENSCFEG0000003614](https://www.ensembl.org/Camelus_ferus/Gene/Summary?g=ENSCFEG00000003614) |
|  |  | *IRF7* | [102520364](https://www.ncbi.nlm.nih.gov/gene/102520364) | [ENSCFEG00000003613](https://www.ensembl.org/Camelus_ferus/Gene/Summary?g=ENS%E6%9E%81%E9%80%9F%E9%A3%9E%E8%89%87%E5%BC%80%E5%A5%96%E7%BB%93%E6%9E%9C%E6%9F%A5%E8%AF%A2%E5%AE%98%E7%BD%910000003613) |
|  |  | *KLRD1* | [102520365](https://www.ncbi.nlm.nih.gov/gene/102520365) | [ENSCFEG00000003612](https://www.ensembl.org/Camelus_ferus/Gene/Summary?g=ENS%E6%9E%81%E9%80%9F%E9%A3%9E%E8%89%87%E5%BC%80%E5%A5%96%E7%BB%93%E6%9E%9C%E6%9F%A5%E8%AF%A2%E5%AE%98%E7%BD%910000003612) |
|  |  | *RTP4* | [102520366](https://www.ncbi.nlm.nih.gov/gene/102520366) | [ENS0000003611](https://www.ensembl.org/Camelus_fer%E6%9E%81%E9%80%9F%E9%A3%9E%E8%89%87%E5%BC%80%E5%A5%96%E7%BB%93%E6%9E%9C%E6%9F%A5%E8%AF%A2%E5%AE%98%E7%BD%91/Gene/Summary?g=ENSCFEG00000003611) |
|  |  | *TNFSF4* | [102520367](https://www.ncbi.nlm.nih.gov/gene/102520367) | [ENS0000003610](https://www.ensembl.org/Camelus_ferus/Gene/Summary?g=ENS%E6%9E%81%E9%80%9F%E9%A3%9E%E8%89%87%E5%BC%80%E5%A5%96%E7%BB%93%E6%9E%9C%E6%9F%A5%E8%AF%A2%E5%AE%98%E7%BD%910000003610) |
|  |  | *TRAT1* | [102520368](https://www.ncbi.nlm.nih.gov/gene/102520368) | [ENS0000003609](https://www.ensembl.org/Camelus_ferus/Gene/Summary?g=ENS%E6%9E%81%E9%80%9F%E9%A3%9E%E8%89%87%E5%BC%80%E5%A5%96%E7%BB%93%E6%9E%9C%E6%9F%A5%E8%AF%A2%E5%AE%98%E7%BD%910000003609) |
|  | *Vicugna pacos* | *GBP5* | [102531361](https://www.ncbi.nlm.nih.gov/gene/102531361) | [ENSVPAG00000003616](https://www.ensembl.org/Vicugna_pacos/Gene/Summary?g=ENSVPAG00000003616) |
|  |  | *GZMB* | [102531362](https://xn--1ts85cy9d12l6d4rn93k52az8qz00a2ukxn0a/www.ncbi.nlm.nih.gov/gene/102531362) | [ENSVPAG00000003615](https://www.ensembl.org/Vicugna_pacos/Gene/Summary?g=ENSVPAG00000003615) |
|  |  | *IFNG* | [102531363](https://www.ncbi.nlm.nih.gov/gene/102531363) | [ENSVPAG00000003614](https://www.ensembl.org/Vicugna_pacos/Gene/Summary?g=ENSVPAG00000003614) |
|  |  | *IRF7* | [102531364](https://www.ncbi.nlm.nih.gov/gene/102531364) | [ENSVPAG00000003613](https://www.ensembl.org/Vicugna_pacos/Gene/Summary?g=ENSVPAG00000003613) |
|  |  | *KLRD1* | [102531365](https://www.ncbi.nlm.nih.gov/gene/102531365) | [ENSVPAG00000003612](https://www.ensembl.org/Vicugna_pacos/Gene/Summary?g=ENSVPAG00000003612) |
|  |  | *RTP* | [102531366](https://www.ncbi.nlm.nih.gov/gene/102531366) | [ENSVPAG00000003611](https://www.ensembl.org/Vicugna_pacos/Gene/Summary?g=ENSVPAG00000003611) |
|  |  | *TNFSF4* | [102531367](https://www.ncbi.nlm.nih.gov/gene/102531367) | [ENSVPAG00000003610](https://www.ensembl.org/Vicugna_pacos/Gene/Summary?g=ENSVPAG00000003610) |
|  |  | *TRAT1* | [102531368](https://www.ncbi.nlm.nih.gov/gene/102531368) | [ENSVPAG00000003609](https://www.ensembl.org/Vicugna_pacos/Gene/Summary?g=ENSVPAG00000003609) |
|  | *Physeter catodon* | *GBP5* | [103087361](https://www.ncbi.nlm.nih.gov/gene/103087361) | [ENSPCAG00000003616](https://www.ensembl.org/Physeter_catodon/Gene/Summary?g=ENSPCAG00000003616) |
|  |  | *GZMB* | [103087362](https://www.ncbi.nlm.nih.gov/gene/%E6%9E%81%E9%80%9F%E9%A3%9E%E8%89%87%E5%BC%80%E5%A5%96%E7%BB%93%E6%9E%9C%E6%9F%A5%E8%AF%A2%E5%AE%98%E7%BD%91) | [ENSPCAG00000003615](https://www.ensembl.org/Physeter_catodon/Gene/Summary?g=ENSPCAG00000003615) |
|  |  | *IFNG* | [103087363](https://www.ncbi.nlm.nih.gov/gene/103087363) | [ENSPCAG00000003614](https://www.ensembl.org/Physeter_catodon/Gene/Summary?g=ENSPCAG00000003614) |
|  |  | *IRF7* | [103087364](https://www.ncbi.nlm.nih.gov/gene/103087364) | [ENSPCAG00000003613](https://www.ensembl.org/Physeter_catodon/Gene/Summary?g=ENSPCAG00000003613) |
|  |  | *KLRD1* | [103087365](https://www.ncbi.nlm.nih.gov/gene/103087365) | [ENSPCAG00000003612](https://www.ensembl.org/Physeter_catodon/Gene/Summary?%E6%9E%81%E9%80%9F%E9%A3%9E%E8%89%87%E5%BC%80%E5%A5%96%E7%BB%93%E6%9E%9C%E6%9F%A5%E8%AF%A2%E5%AE%98%E7%BD%91=ENSPCAG00000003612) |
|  |  | *RTP4* | [103087366](https://www.ncbi.nlm.nih.gov/gene/103087366) | [ENSPCAG00000003611](https://www.ensembl.org/Physeter_catodon/Gene/Summary?g=ENSPCAG00000003611) |
|  |  | *TNFSF4* | [103087367](https://www.ncbi.nlm.nih.gov/gene/103087367) | [ENSPCAG00000003610](https://www.ensembl.org/Physeter_catodon/Gene/Summary?g=ENSPCAG00000003610) |
|  |  | *TRAT1* | [103087368](https://www.ncbi.nlm.nih.gov/gene/103087368) | [ENSPCAG00000003609](https://www.ensembl.org/Physeter_catodon/Gene/Summary?g=ENSPCAG00000003609) |
|  | *Orcinus orca* | *GBP5* | [101263361](https://www.ncbi.nlm.nih.gov/gene/101263361) | [ENSOORG00000003616](https://www.ensembl.org/Orcinus_orca/Gene/Summary?g=ENSOORG00000003616) |
|  |  | *GZMB* | [101263362](https://www.ncbi.nlm.nih.gov/gene/101263362) | [ENSOORG00000003615](https://www.ensembl.org/Orcinus_orca/Gene/Summary?g=ENSOORG00000003615) |
|  |  | *IFNG* | [101263363](https://www.ncbi.nlm.nih.gov/gene/101263363) | [ENSOORG00000003614](https://www.ensembl.org/Orcinus_orca/Gene/Summary?g=ENSOORG00000003614) |
|  |  | *IRF7* | [101263364](https://www.ncbi.nlm.nih.gov/gene/101263364) | [ENSOORG00000003613](https://www.ensembl.org/Orcinus_orca/Gene/Summary?g=ENSOORG00000003613) |
|  |  | *KLRD1* | [101263365](https://www.ncbi.nlm.nih.gov/gene/101263365) | [ENSOORG00000003612](https://www.ensembl.org/Orcinus_orca/Gene/Summary?g=ENSOORG00000003612) |
|  |  | *RTP4* | [101263366](https://www.ncbi.nlm.nih.gov/gene/101263366) | [ENSOORG00000003611](https://www.ensembl.org/Orcinus_orca/Gene/Summary?g=ENSOORG00000003611) |
|  |  | *TNFSF4* | [101263367](https://www.ncbi.nlm.nih.gov/gene/101263367%E6%9E%81%E9%80%9F%E9%A3%9E%E8%89%87%E5%BC%80%E5%A5%96%E7%BB%93%E6%9E%9C%E6%9F%A5%E8%AF%A2%E5%AE%98%E7%BD%91) | [ENSOORG00000003610](https://www.ensembl.org/Orcinus_orca/Gene/Summary?g=ENSOORG00000003610) |
|  |  | *TRAT1* | [101263368](https://www.ncbi.nlm.nih.gov/gene/101263368) | [ENSOORG00000003609](https://www.ensembl.org/Orcinus_orca/Gene/Summary?g=ENSOORG00000003609) |
| Carnivora | *Canis lupus familiaris* | *GBP5* | [403622](https://www.ncbi.nlm.nih.gov/gene/403622) | [ENSCAFG00000003616](https://www.ensembl.org/Canis_lupus_familiaris/Gene/Summary?g=ENSCAFG00000003616) |
|  |  | *GZMB* | [403623](https://www.ncbi.nlm.nih.gov/gene/403623) | [ENSCAFG00000003615](https://www.ensembl.org/Canis_lupus_familiaris/Gene/Summary?g=ENSCAFG00000003615) |
|  |  | *IFNG* | [403624](https://www.ncbi.nlm.nih.gov/gene/403624) | [ENSCAFG00000003614](https://www.ensembl.org/Canis_lupus_familiaris/Gene/Summary?g=ENSCAFG00000003614) |
|  |  | *IRF7* | [403625](https://www.ncbi.nlm.nih.gov/gene/403625) | [ENSCAFG00000003613](https://www.ensembl.org/Canis_lupus_familiaris/Gene/Summary?g=ENSCAFG%E6%9E%81%E9%80%9F%E9%A3%9E%E8%89%87%E5%BC%80%E5%A5%96%E7%BB%93%E6%9E%9C%E6%9F%A5%E8%AF%A2%E5%AE%98%E7%BD%910000003613) |
|  |  | *KLRD1* | [403626](https://www.ncbi.nlm.nih.gov/gene/403626) | [ENSCAFG00000003612](https://www.ensembl.org/Canis_lupus_familiaris/Gene/Summary?g=ENSCAFG00000003612) |
|  |  | *RTP4* | [403627](https://www.ncbi.nlm.nih.gov/gene/403627) | [ENSCAFG00000003611](https://www.ensembl.org/Canis_lupus_familiaris/Gene/Summary?g=ENSCAFG00000003611) |
|  |  | *TNFSF4* | [403628](https://www.ncbi.nlm.nih.gov/gene/403628) | [ENSCAFG00000003610](https://www.ensembl.org/Canis_lupus_familiaris/Gene/Summary?g=ENSCAFG00000003610) |
|  |  | *TRAT1* | [403629](https://www.ncbi.nlm.nih.gov/gene/403629) | [ENSCAFG00000003609](https://www.ensembl.org/Canis_lupus_familiaris/Gene/Summary?g=ENSCAFG00000003609) |
|  | *Felis catus* | *GBP5* | [101086361](https://www.ncbi.nlm.nih.gov/gene/101086361) | [ENSFCAG00000003616](https://www.ensembl.org/Felis_catus/Gene/Summary?g=ENSFCAG00000003616) |
|  |  | *GZMB* | [101086362](https://www.ncbi.nlm.nih.gov/gene/101086362) | [ENSFCAG00000003615](https://www.ensembl.org/Felis_catus/Gene/Summary?g=ENSFCAG00000003615) |
|  |  | *IFNG* | [101086363](https://www.ncbi.nlm.nih.gov/gene/101086363) | [ENSFCAG00000003614](https://www.ensembl.org/Felis_catus/Gene/Summary?g=ENSFCAG00000003614) |
|  |  | *IRF7* | [101086364](https://www.ncbi.nlm.nih.gov/gene/101086364) | [ENSFCAG00000003613](https://www.ensembl.org/Felis_catus/Gene/Summary?g=ENSFCAG00000003613) |
|  |  | *KLRD1* | [101086365](https://www.ncbi.nlm.nih.gov/gene/101086365) | [ENSFCAG00000003612](https://www.ensembl.org/Felis_catus/Gene%E6%9E%81%E9%80%9F%E9%A3%9E%E8%89%87%E5%BC%80%E5%A5%96%E7%BB%93%E6%9E%9C%E6%9F%A5%E8%AF%A2%E5%AE%98%E7%BD%91/Summary?g=ENSFCAG00000003612) |
|  |  | *RTP4* | [101086366](https://www.ncbi.nlm.nih.gov/gene/101086366) | [ENSFCAG00000003611](https://www.ensembl.org/Felis_catus/Gene/Summary?g=ENSFCAG00000003611) |
|  |  | *TNFSF4* | [101086367](https://www.ncbi.nlm.nih.gov/gene/101086367) | [ENSFCAG00000003610](https://www.ensembl.org/Felis_catus/Gene/Summary?g=ENSFCAG00000003610) |
|  |  | *TRAT1* | [101086368](https://www.ncbi.nlm.nih.gov/gene/101086368) | [ENSFCAG00000003609](https://www.ensembl.org/Felis_catus/Gene/Summary?g=ENSFCAG00000003609) |
|  | *Ailuropoda melanoleuca* | *GBP5* | [100469361](https://www.ncbi.nlm.nih.gov/gene/100469361) | [ENSAMEG00000003616](https://www.ensembl.org/Ailuropoda_melanoleuca/Gene/Summary?g=ENSAMEG00000003616) |
|  |  | *GZMB* | [100469362](https://www.ncbi.nlm.nih.gov/gene/100469362) | [ENSAMEG00000003615](https://www.ensembl.org/Ailuropoda_melanoleuca/Gene/Summary?g=ENSAMEG00000003615) |
|  |  | *IFNG* | [100469363](https://www.ncbi.nlm.nih.gov/gene/100469363) | [ENSAMEG00000003614](https://www.ensembl.org/Ailuropoda_melanoleuca/Gene/Summary?g=ENSAMEG00000003614) |
|  |  | *IRF7* | [100469364](https://www.ncbi.nlm.nih.gov/gene/100469364) | [ENSAMEG00000003613](https://www.ensembl.org/Ailuropoda_melanoleuca/Gene/Summary?g=ENSAMEG00000003613) |
|  |  | *KLRD1* | [100469365](https://www.ncbi.nlm.nih.gov/gene/100469365) | [ENSAMEG00000003612](https://www.ensembl.org/Ailuropoda_melanoleuca/Gene/Summary?g=ENSAMEG00000003612) |
|  |  | *RTP4* | [100469366](https://www.ncbi.nlm.nih.gov/gene/100469366) | [ENSAMEG00000003611](https://www.ensembl.org/Ailuropoda_melanoleuca/Gene/Summary?g=ENSAMEG00000003611) |
|  |  | *TNFSF4* | [100469367](https://www.ncbi.nlm.nih.gov/gene/100469367) | [ENSAMEG00000003610](https://www.ensembl.org/Ailuropoda_melanoleuca/Gene/Summary?g=ENSAMEG00000003610) |
|  |  | *TRAT1* | [100469368](https://www.ncbi.nlm.nih.gov/gene/100469368) | [ENSAMEG00000003609](https://www.ensembl.org/Ailuropoda_melanoleuca/Gene/Summary?g=ENSAMEG00000003609) |
|  | *Mustela putorius furo* | *GBP5* | [101826361](https://www.ncbi.nlm.nih.gov/gene/101826361) | [ENSMPUG00000003616](https://www.ensembl.org/Mustela_putorius_furo/G%E6%9E%81%E9%80%9F%E9%A3%9E%E8%89%87%E5%BC%80%E5%A5%96%E7%BB%93%E6%9E%9C%E6%9F%A5%E8%AF%A2%E5%AE%98%E7%BD%91/Summary?g=ENSMPUG00000003616) |
|  |  | *GZMB* | [101826362](https://www.ncbi.nlm.nih.gov/gene/101826362) | [ENSMPUG00000003615](https://www.ensembl.org/Mustela_putorius_furo/Gene/Summary?g=ENSMPUG00000003615) |
|  |  | *IFNG* | [101826363](https://www.ncbi.nlm.nih.gov/gene/101826363) | [ENSMPUG00000003614](https://www.ensembl.org/Mustela_putorius_furo/Gene/Summary?g=ENSMPUG00000003614) |
|  |  | *KLRD1* | [101826365](https://www.ncbi.nlm.nih.gov/gene/101826365) | [ENSMPUG00000003612](https://www.ensembl.org/Mustela_putorius_furo/Gene/Summary?g=ENSMPUG00000003612) |
|  |  | *RTP4* | [101826366](https://www.ncbi.nlm.nih.gov/gene/101826366) | [ENSMPUG00000003611](https://www.ensembl.org/Mustela_putorius_furo/Gene/Summary?g=ENSMPUG00000003611) |
|  |  | *TNFSF4* | [101826367](https://www.ncbi.nlm.nih.gov/gene/101826367) |  |
|  |  | *TRAT1* | [101826368](https://www.ncbi.nlm.nih.gov/gene/101826368) | [ENSMPUG00000003609](https://www.ensembl.org/Mustela_putorius_furo/Gene/Summary?g=ENSMPUG00000003609) |
|  | *Enhydra lutris* | *GBP5* | [101927361](https://www.ncbi.nlm.nih.gov/gene/101927361) | [ENSELUG00000003616](https://www.ensembl.org/Enhydra_lutris/Gene/Summary?g=ENSELUG00000003616) |
|  |  | *GZMB* | [101927362](https://www.ncbi.nlm.nih.gov/gene/101927362) | [ENSELUG00000003615](https://www.ensembl.org/Enhydra_lutris/Gene/Summary?g=ENSELUG00000003615) |
|  |  | *IFNG* | [101927363](https://www.ncbi.nlm.nih.gov/gene/101927363) | [ENSELUG00000003614](https://www.ensembl.org/Enhydra_lutris/Gene/Summary?g=ENSELUG00000003614) |
|  |  | *IRF7* | [101927364](https://www.ncbi.nlm.nih.gov/gene/101927364) | [ENSELUG00000003613](https://www.ensembl.org/Enhydra_lutris/Gene/Summary?g=ENSELUG00000003613) |
|  |  | *KLRD1* | [101927](https://www.ncbi.nlm.nih.gov/gene/101927365) | [ENSELUG00000003612](https://www.ensembl.org/Enhydra_lutris/Gene/Summary?g=ENSELUG00000003612) |
|  |  | *RTP4* | [101927366](https://www.ncbi.nlm.nih.gov/gene/101927366) | [ENSELUG00000003611](https://www.ensembl.org/Enhydra_lutris/Gene/Summary?g=ENSELUG00000003611) |
|  |  | *TNFSF4* | [101927367](https://www.ncbi.nlm.nih.gov/gene/101927367) | [ENSELUG00000003610](https://www.ensembl.org/Enhydra_lutris/Gene/Summary?g=ENSELUG00000003610) |
|  |  | *TRAT1* | [101927368](https://www.ncbi.nlm.nih.gov/gene/101927368) | [ENSELUG00000003609](https://www.ensembl.org/Enhydra_lutris/Gene/Summary?g=ENSELUG00000003609) |
| Chiroptera | *Pteropus vampyrus* | *GBP5* | [101508361](https://www.ncbi.nlm.nih.gov/gene/101508361) | [ENSPVAG00000003616](https://www.ensembl.org/Pteropus_vampyrus/Gene/Summary?g=ENSPVAG00000003616) |
|  |  | *GZMB* | [101508362](https://www.ncbi.nlm.nih.gov/gene/101508362) | [ENSPVAG00000003615](https://www.ensembl.org/Pteropus_vampyrus/Gene/Summary?g=ENSPVAG00000003615) |
|  |  | *IFNG* | [101508363](https://www.ncbi.nlm.nih.gov/gene/101508363) | [ENSPVAG00000003614](https://www.ensembl.org/Pteropus_vampyrus/Gene/Summary?g=ENSPVAG00000003614) |
|  |  | *IRF7* | [101508364](https://www.ncbi.nlm.nih.gov/gene/101508364) | [ENSPVAG00000003613](https://www.ensembl.org/Pteropus_vampyrus/Gene/Summary?g=ENSPVAG00000003613) |
|  |  | *KLRD1* | [101508365](https://www.ncbi.nlm.nih.gov/gene/101508365) | [ENSPVAG00000003612](https://xn--www-gj8et3hp3gn9qigajyk84pyeby5trr6a2nmu35a.ensembl.org/Pteropus_vampyrus/Gene/Summary?g=ENSPVAG00000003612) |
|  |  | *RTP4* | [101508366](https://www.ncbi.nlm.nih.gov/gene/101508366) | [ENSPVAG00000003611](https://www.ensembl.org/Pteropus_vampyrus/Gene/Summary?g=ENSPVAG00000003611) |
|  |  | *TNFSF4* | [101508367](https://www.ncbi.nlm.nih.gov/gene/101508367) | [ENSPVAG00000003610](https://www.ensembl.org/Pteropus_vampyrus/Gene/Summary?g=ENSPVAG00000003610) |
|  |  | *TRAT1* | [101508368](https://www.ncbi.nlm.nih.gov/gene/101508368) |  |
|  | *Rousettus aegyptiacus* | *GBP5* | [101609361](https://www.ncbi.nlm.nih.gov/gene/101609361) | [ENSRAEG00000003616](https://www.ensembl.org/Rousettus_aegyptiacus/Gene/Summary?g=ENSRAEG00000003616) |
|  |  | *GZMB* | [101609362](https://www.ncbi.nlm.nih.gov/gene/101609362) | [ENSRAEG00000003615](https://www.ensembl.org/Rousettus_aegyptiacus/Gene/Summary?g=ENSRAEG00000003615) |
|  |  | *IFNG* | [101609363](https://www.ncbi.nlm.nih.gov/gene/101609363) | [ENSRAEG00000003614](https://www.ensembl.org/Rousettus_aegyptiacus/Gene/Summary?g=ENSRAEG00000003614) |
|  |  | *IRF7* | [101609364](https://www.ncbi.nlm.nih.gov/gene/101609364) | [ENSRAEG00000003613](https://www.ensembl.org/Rousettus_aegyptiacus/Gene/Summary?g=ENSRAEG00000003613%E6%9E%81%E9%80%9F%E9%A3%9E%E8%89%87%E5%BC%80%E5%A5%96%E7%BB%93%E6%9E%9C%E6%9F%A5%E8%AF%A2%E5%AE%98%E7%BD%91) |
|  |  | *KLRD1* | [101609365](https://www.ncbi.nlm.nih.gov/gene/101609365) | [ENSRAEG00000003612](https://www.ensembl.org/Rousettus_aegyptiacus/Gene/Summary?g=ENSRAEG00000003612) |
|  |  | *RTP4* | [101609366](https://www.ncbi.nlm.nih.gov/gene/101609366) | [ENSRAEG00000003611](https://www.ensembl.org/Rousettus_aegyptiacus/Gene/Summary?g=ENSRAEG00000003611) |
|  |  | *TNFSF4* | [101609367](https://www.ncbi.nlm.nih.gov/gene/101609367) | [ENSRAEG00000003610](https://www.ensembl.org/Rousettus_aegyptiacus/Gene/Summary?g=ENSRAEG00000003610) |
|  |  | *TRAT1* | [101609368](https://www.ncbi.nlm.nih.gov/gene/101609368) | [ENSRAEG00000003609](https://www.ensembl.org/Rousettus_aegyptiacus/Gene/Summary?g=ENSRAEG00000003609) |
|  | *Myotis lucifugus* | *GBP5* | [101710361](https://www.ncbi.nlm.nih.gov/gene/101710361) | [ENSMLUG00000003616](https://www.ensembl.org/Myotis_lucifugus/Gene/Summary?g=ENSMLUG00000003616) |
|  |  | *GZMB* | [101710362](https://www.ncbi.nlm.nih.gov/gene/101710362) | [ENSMLUG00000003615](https://www.ensembl.org/Myotis_luc%E6%9E%81%E9%80%9F%E9%A3%9E%E8%89%87%E5%BC%80%E5%A5%96%E7%BB%93%E6%9E%9C%E6%9F%A5%E8%AF%A2%E5%AE%98%E7%BD%91/Gene/Summary?g=ENSMLUG00000003615) |
|  |  | *IFNG* | [101710363](https://www.ncbi.nlm.nih.gov/gene/101710363) | [ENSMLUG00000003614](https://www.ensembl.org/Myotis_lucifugus/Gene/Summary?g=ENSMLUG00000003614) |
|  |  | *IRF7* | [101710364](https://www.ncbi.nlm.nih.gov/gene/101710364) | [ENSMLUG00000003613](https://www.ensembl.org/Myotis_lucifugus/Gene/Summary?g=ENSMLUG00000003613) |
|  |  | *KLRD1* | [101710365](https://www.ncbi.nlm.nih.gov/gene/101710365) | [ENSMLUG00000003612](https://www.ensembl.org/Myotis_lucifugus/Gene/Summary?g=ENSMLUG00000003612) |
|  |  | *RTP4* | [101710366](https://www.ncbi.nlm.nih.gov/gene/101710366) | [ENSMLUG00000003611](https://www.ensembl.org/Myotis_lucifugus/Gene/Summary?g=ENSMLUG00000003611) |
|  |  | *TNFSF4* | [101710367](https://www.ncbi.nlm.nih.gov/gene/101710367) | [ENSMLUG00000003610](https://www.ensembl.org/Myotis_lucifugus/Gene/Summary?g=ENSMLUG00000003610) |
|  |  | *TRAT1* | [101710368](https://www.ncbi.nlm.nih.gov/gene/101710368) | [ENSMLUG00000003609](https://www.ensembl.org/Myotis_lucifug%E6%9E%81%E9%80%9F%E9%A3%9E%E8%89%87%E5%BC%80%E5%A5%96%E7%BB%93%E6%9E%9C%E6%9F%A5%E8%AF%A2%E5%AE%98%E7%BD%91/Gene/Summary?%E6%9E%81%E9%80%9F%E9%A3%9E%E8%89%87%E5%BC%80%E5%A5%96%E7%BB%93%E6%9E%9C%E6%9F%A5%E8%AF%A2%E5%AE%98%E7%BD%91=ENSMLUG00000003609) |
|  | *Myotis brandtii* | *GBP5* | [101811361](https://www.ncbi.nlm.nih.gov/gene/101811361) | [ENSMBRG00000003616](https://www.ensembl.org/Myotis_brandtii/Gene/Summary?g=ENSMBRG00000003616) |
|  |  | *GZMB* | [101811362](https://www.ncbi.nlm.nih.gov/gene/101811362) | [ENSMBRG00000003615](https://www.ensembl.org/Myotis_brandtii/Gene/Summary?g=ENSMBRG%E6%9E%81%E9%80%9F%E9%A3%9E%E8%89%87%E5%BC%80%E5%A5%96%E7%BB%93%E6%9E%9C%E6%9F%A5%E8%AF%A2%E5%AE%98%E7%BD%910000003615) |
|  |  | *IFNG* | [101811363](https://www.ncbi.nlm.nih.gov/gene/101811363) | [ENSMBRG00000003614](https://www.ensembl.org/Myotis_brandtii/Gene/Summary?g=ENSMBRG00000003614) |
|  |  | *IRF7* | [101811364](https://www.ncbi.nlm.nih.gov/gene/101811364) | [ENSMBRG00000003613](https://www.ensembl.org/Myotis_brandtii/Gene/Summary?g=ENSMBRG00000003613) |
|  |  | *KLRD1* | [101811365](https://www.ncbi.nlm.nih.gov/gene/101811365) | [ENSMBRG00000003612](https://www.ensembl.org/Myotis_brandtii/Gene/Summary?g=ENSMBRG00000003612) |
|  |  | *RTP4* | [101811366](https://www.ncbi.nlm.nih.gov/gene/101811366) | [ENSMBRG00000003611](https://www.ensembl.org/Myotis_brandtii/Gene/Summary?g=ENSMBRG00000003611) |
|  |  | *TNFSF4* | [101811367](https://www.ncbi.nlm.nih.gov/gene/101811367) | [ENSMBRG00000003610](https://www.ensembl.org/Myotis_brandtii/Gene/Summary?g=ENSMBRG00000003610) |
|  |  | *TRAT1* | [101811368](https://www.ncbi.nlm.nih.gov/gene/101811368) | [ENSMBRG00000003609](https://www.ensembl.org/Myotis_brandtii/Gene/Summary?g=ENSMBRG00000003609) |
| Perissodactyla | *Equus caballus* | *GBP5* | [100064361](https://www.ncbi.nlm.nih.gov/gene/100064361) | [ENSECAG00000003616](https://www.ensembl.org/Equus_caballus/Gene/Summary?g=ENSECAG00000003616) |
|  |  | *GZMB* | [100064362](https://www.ncbi.nlm.nih.gov/gene/100064362) | [ENSECAG00000003615](https://www.ensembl.org/Equus_caballus/Gene/Summary?g=ENSECAG00000003615) |
|  |  | *IFNG* | [100064363](https://www.ncbi.nlm.nih.gov/gene/100064363) | [ENSECAG00000003614](https://www.ensembl.org/Equus_caballus/Gene/Summary?g=ENSECAG00000003614) |
|  |  | *IR* | [100064364](https://www.ncbi.nlm.nih.gov/gene/100064364) | [ENSECAG00000003613](https://www.ensembl.org/Equus_caballus/Gene/Summary?g=ENSECAG00000003613) |
|  |  | *KLRD1* | [100064365](https://www.ncbi.nlm.nih.gov/gene/100064365%E6%9E%81%E9%80%9F%E9%A3%9E%E8%89%87%E5%BC%80%E5%A5%96%E7%BB%93%E6%9E%9C%E6%9F%A5%E8%AF%A2%E5%AE%98%E7%BD%91) | [ENSECAG00000003612](https://www.ensembl.org/Equus_caballus/Gene/Summary?g=ENSECAG000000036%E6%9E%81%E9%80%9F%E9%A3%9E%E8%89%87%E5%BC%80%E5%A5%96%E7%BB%93%E6%9E%9C%E6%9F%A5%E8%AF%A2%E5%AE%98%E7%BD%91) |
|  |  | *RTP4* | [100064366](https://www.ncbi.nlm.nih.gov/gene/100064366) | [ENSECAG00000003611](https://www.ensembl.org/Equus_caballus/Gene/Summary?g=ENSECAG00000003611) |
|  |  | *TNFSF4* | [100064367](https://www.ncbi.nlm.nih.gov/gene/100064367) | [ENSECAG00000003610](https://www.ensembl.org/Equus_caballus/Gene/Summary?g=ENSECAG00000003610) |
|  |  | *TRAT1* | [100064368](https://www.ncbi.nlm.nih.gov/gene/100064368) | [ENSECAG00000003609](https://www.ensembl.org/Equus_caballus/Gene/Summary?g=ENSECAG00000003609) |
|  | *Ceratotherium simum* | *GBP5* | [101912361](https://www.ncbi.nlm.nih.gov/gene/101912361) | [ENSCSIG00000003616](https://www.ensembl.org/Ceratotherium_simum/Gene/Summary?g=ENSCSIG00000003616) |
|  |  | *GZMB* | [101912362](https://www.ncbi.nlm.nih.gov/gene/101912362) | [ENSCSIG00000003615](https://www.ensembl.org/Ceratotherium_simum/Gene/Summary?g=ENSCSIG00000003615) |
|  |  | *IFNG* | [101912363](https://www.ncbi.nlm.nih.gov/gene/101912363) | [ENSCSIG00000003614](https://www.ensembl.org/Ceratotherium_simum/Gene/Summary?g=ENSCSIG00000003614) |
|  |  | *IRF7* | [101912364](https://www.ncbi.nlm.nih.gov/gene/101912364) | [ENSCSIG00000003613](https://www.ensembl.org/Ceratotherium_simum/Gene/Summary?g=ENSCSIG00000003613) |
|  |  | *KLRD1* | [101912365](https://www.ncbi.nlm.nih.gov/gene/101912365) | [ENSCSIG00000003612](https://www.ensembl.org/Ceratotherium_simum/Gene/Summary?g=ENSCSIG00000003612) |
|  |  | *RTP4* | [101912366](https://www.ncbi.nlm.nih.gov/gene/101912366) | [ENSCSIG00000003611](https://www.ensembl.org/Ceratotherium_simum/Gene/Summary?g=ENSCSIG00000003611) |
|  |  | *TNFSF4* | [101912367](https://www.ncbi.nlm.nih.gov/g%E6%9E%81%E9%80%9F%E9%A3%9E%E8%89%87%E5%BC%80%E5%A5%96%E7%BB%93%E6%9E%9C%E6%9F%A5%E8%AF%A2%E5%AE%98%E7%BD%91/101912367) | [ENSCSIG00000003610](https://www.ensembl.org/Ceratotherium_simum/Gene/Summary?g=ENSCSIG00000003610) |
|  |  | *TRAT1* | [101912368](https://www.ncbi.nlm.nih.gov/gene/101912368) | [ENSCSIG00000003609](https://www.ensembl.org/Ceratotherium_simum/Gene/Summary?g=ENSCSIG00000003609) |
| Lagomorpha | *Oryctolagus cuniculus* | *GBP5* | [100009361](https://www.ncbi.nlm.nih.gov/gene/100009361) | [ENSOCUG00000003616](https://www.ensembl.org/Oryctolagus_cuniculus/Gene/Summary?g=ENSOCUG00000003616) |
|  |  | *GZMB* | [100009362](https://www.ncbi.nlm.nih.gov/gene/100009362) | [ENSOCUG00000003615](https://www.ensembl.org/Oryctolagus_cuniculus/Gene/Summary?g=ENSOCUG00000003615) |
|  |  | *IFNG* | [100009363](https://www.ncbi.nlm.nih.gov/gene/100009363) | [ENSOCUG00000003614](https://www.ensembl.org/Oryctolagus_cuniculus/Gene/Summary?g=ENSOCUG00000003614) |
|  |  | *IRF7* | [100009364](https://www.ncbi.nlm.nih.gov/gene/100009364) | [ENSOCUG00000003613](https://www.ensembl.org/Oryctolagus_cuniculus/Gene/Summary?g=ENSOCUG00000003613) |
|  |  | *KLRD1* | [100009365](https://www.ncbi.nlm.nih.gov/gene/100009365) | [ENSOCUG00000003612](https://www.ensembl.org/Oryctolagus_cuniculus/Gene/Summary?g=ENSOCUG00000003612) |
|  |  | *RTP4* | [100009366](https://www.ncbi.nlm.nih.gov/gene/100009366) | [ENSOCUG00000003611](https://www.ensembl.org/Oryctolagus_cuniculus/Gene/Summary?g=ENSOCUG00000003611) |
|  |  | *TNFSF4* | [100009367](https://www.ncbi.nlm.nih.gov/gene/100009367) | [ENSOCUG00000003610](https://www.ensembl.org/Oryctolagus_cuniculus/Gene/Summary?g=ENSOCUG00000003610) |
|  |  | *TRAT1* | [100009368](https://www.ncbi.nlm.nih.gov/gene/100009368) | [ENSOCUG00000003609](https://www.ensembl.org/Oryctolagus_cuniculus/Gene/Summary?g=ENSOCUG00000003609) |
| Eulipotyphla | *Sorex araneus* | *GBP5* | [101713361](https://www.ncbi.nlm.nih.gov/gene/101713361) | [ENSSAAG00000003616](https://www.ensembl.org/Sorex_araneus/Gene/Summary?g=ENSSAAG00000003616) |
|  |  | *GZMB* | [101713362](https://www.ncbi.nlm.nih.gov/gene/101713362) | [ENSSAAG00000003615](https://www.ensembl.org/Sore%E6%9E%81%E9%80%9F%E9%A3%9E%E8%89%87%E5%BC%80%E5%A5%96%E7%BB%93%E6%9E%9C%E6%9F%A5%E8%AF%A2%E5%AE%98%E7%BD%91/Gene/Summary?g=ENSSAAG00000003615) |
|  |  | *IFNG* | [101713363](https://www.ncbi.nlm.nih.gov/gene/101%E6%9E%81%E9%80%9F%E9%A3%9E%E8%89%87%E5%BC%80%E5%A5%96%E7%BB%93%E6%9E%9C%E6%9F%A5%E8%AF%A2%E5%AE%98%E7%BD%91) | [ENSSAAG00000003614](https://www.ensembl.org/Sorex_araneus/Gene/Summary?g=ENSSAAG00000003614) |
|  |  | *IRF7* | [101713364](https://www.ncbi.nlm.nih.gov/gene/101713364) | [ENSSAAG00000003613](https://www.ensembl.org/Sorex_araneus/Gene/Summary?g=ENSSAAG00000003613) |
|  |  | *KLRD1* | [101713365](https://www.ncbi.nlm.nih.gov/gene/101713365) | [ENSSAAG00000003612](https://www.ensembl.org/Sorex_araneus/Gene/Summary?g=ENSSAAG00000003612) |
|  |  | *RTP4* | [101713366](https://www.ncbi.nlm.nih.gov/gene/101713366) | [ENSSAAG00000003611](https://www.ensembl.org/Sorex_araneus/Gene/Summary?g=ENSSAAG00000003611) |
|  |  | *TNFSF4* | [101713367](https://www.ncbi.nlm.nih.gov/gene/101713367) | [ENSSAAG00000003610](https://www.ensembl.org/Sorex_araneus/Gene/Summary?g=ENSSAAG00000003610) |
|  |  | *TRAT1* | [101713368](https://www.ncbi.nlm.nih.gov/gene/101713368) | [ENSSAAG00000003609](https://www.ensembl.org/Sorex_araneus/Gene/Summary?g=ENSSAAG00000003609) |
|  | *Erinaceus europaeus* | *GBP5* | [101814361](https://www.ncbi.nlm.nih.gov/gene/101814361) | [ENSEEUG00000003616](https://www.ensembl.org/Erinaceus_europaeus/Gene/Summary?g=ENSEEUG00000003616) |
|  |  | *GZMB* | [101814362](https://www.ncbi.nlm.nih.gov/gene/101814362) | [ENSEEUG00000003615](https://www.ensembl.org/Erinaceus_europaeus/Gene/Summary?g=ENSEEUG00000003615) |
|  |  | *IFNG* | [101814363](https://www.ncbi.nlm.nih.gov/gene/101814363) | [ENSEEUG00000003614](https://www.ensembl.org/Erinaceus_europaeus/Gene/Summary?g=ENSEEUG00000003614) |
|  |  | *IRF7* | [101814364](https://www.ncbi.nlm.nih.gov/gene/101814364) | [ENSEEUG00000003613](https://www.ensembl.org/Erinaceus_europaeus/Gene/Summary?g=ENSEEUG00000003613) |
|  |  | *KLRD1* | [101814365](https://www.ncbi.nlm.nih.gov/gene/101814365) | [ENSEEUG00000003612](https://www.ensembl.org/Erinaceus_europaeus/Gene/Summary?g=ENSEEUG00000003612) |
|  |  | *RTP4* | [101814366](https://www.ncbi.nlm.nih.gov/gene/101814366) | [ENSEEUG00000003611](https://www.ensembl.org/Erinaceus_europaeus/Gene/Summary?g=ENSEEUG00000003611) |
|  |  | *TNFSF4* | [101814367](https://www.ncbi.nlm.nih.gov/gene/101814367) | [ENSEEUG00000003610](https://www.ensembl.org/Erinaceus_europaeus/Gene/Summary?g=ENSEEUG00000003610) |
|  |  | *TRAT1* | [101814368](https://www.ncbi.nlm.nih.gov/gene/101814368) | [ENSEEUG00000003609](https://www.ensembl.org/Erinaceus_europaeus/Gene/Summary?g=ENSEEUG%E6%9E%81%E9%80%9F%E9%A3%9E%E8%89%87%E5%BC%80%E5%A5%96%E7%BB%93%E6%9E%9C%E6%9F%A5%E8%AF%A2%E5%AE%98%E7%BD%910000003609) |
| Didelphimorphia | *Monodelphis domestica* | *GBP5* | [100023361](https://www.ncbi.nlm.nih.gov/gene/100023361) | [ENSMDOG00000003616](https://www.ensembl.org/Monodelphis_domestica/Gene/Summary?g=ENSMDOG00000003616) |
|  |  | *GZMB* | [100023362](https://www.ncbi.nlm.nih.gov/gene/100023362) | [ENSMDOG00000003615](https://www.ensembl.org/Monodelphis_domestica/Gene/Summary?g=ENSMDOG00000003615) |
|  |  | *IFNG* | [100023363](https://www.ncbi.nlm.nih.gov/gene/100023363) | [ENSMDOG00000003614](https://www.ensembl.org/Monodelphis_domestica/Gene/Summary?g=ENSMDOG00000003614) |
|  |  | *IRF7* | [100023364](https://www.ncbi.nlm.nih.gov/gene/100023364) |  |
|  |  | *KLRD1* | [100023365](https://www.ncbi.nlm.nih.gov/gene/100023365) | [ENSMDOG00000003612](https://www.ensembl.org/Monodelphis_domestica/Gene/Summary?g=ENSMDOG00000003612) |
|  |  | *RTP4* | [100023366](https://www.ncbi.nlm.nih.gov/gene/100023366) | [ENSMDOG00000003611](https://www.ensembl.org/Monodelphis_domestica/Gene/Summary?g=ENSMDOG00000003611) |
|  |  | *TNFSF4* | [100023367](https://www.ncbi.nlm.nih.gov/gene/100023367) | [ENSMDOG00000003610](https://www.ensembl.org/Monodelphis_domestica/Gene/Summary?g=ENSMDOG00000003610) |
|  |  | *TRAT1* | [100023368](https://www.ncbi.nlm.nih.gov/gene/100023368) | [ENSMDOG00000003609](https://www.ensembl.org/Monodelphis_domestica/Gene/Summary?g=ENSMDOG00000003609) |
| Afrotheria | *Loxodonta africana* | *GBP5* | [100734361](https://www.ncbi.nlm.nih.gov/gene/100734361) | [ENSLAFG00000003616](https://www.ensembl.org/Loxodonta_africana/Gene/Summary?g=ENSLAFG00000003616) |
|  |  | *GZMB* | [100734362](https://www.ncbi.nlm.nih.gov/gene/100734362) | [ENSLAFG00000003615](https://www.ensembl.org/Loxodonta_africana/Gene/%E6%9E%81%E9%80%9F%E9%A3%9E%E8%89%87%E5%BC%80%E5%A5%96%E7%BB%93%E6%9E%9C%E6%9F%A5%E8%AF%A2%E5%AE%98%E7%BD%91?g=ENSLAFG00000003615) |
|  |  | *IFNG* | [100734363](https://www.ncbi.nlm.nih.gov/gene/100734363) | [ENSLAFG00000003614](https://www.ensembl.org/Loxodonta_africana/Gene/Summary?g=ENSLAFG00000003614) |
|  |  | *IRF7* | [100734364](https://www.ncbi.nlm.nih.gov/gene/100734364) | [ENSLAFG00000003613](https://www.ensembl.org/Loxodonta_africana/Gene/Summary?g=ENSLAFG00000003613) |
|  |  | *KLRD1* | [100734365](https://www.ncbi.nlm.nih.gov/g%E6%9E%81%E9%80%9F%E9%A3%9E%E8%89%87%E5%BC%80%E5%A5%96%E7%BB%93%E6%9E%9C%E6%9F%A5%E8%AF%A2%E5%AE%98%E7%BD%91/100734365) | [ENSLAFG00000003612](https://www.ensembl.org/Loxodonta_africana/Gene/Summary?g=ENSLAFG00000003612) |
|  |  | *RTP4* | [100734366](https://www.ncbi.nlm.nih.gov/gene/100734366) | [ENSLAFG00000003611](https://www.ensembl.org/Loxodonta_africana/Gene/Summary?g=ENSLAFG00000003611) |
|  |  | *TNFSF4* | [100734367](https://www.ncbi.nlm.nih.gov/gene/100734367) | [ENSLAFG00000003610](https://www.ensembl.org/Loxodonta_africana/Gene/Summary?g=ENSLAFG00000003610) |
|  |  | *TRAT1* | [100734368](https://www.ncbi.nlm.nih.gov/gene/100734368) | [ENSLAFG00000003609](https://www.ensembl.org/L%E6%9E%81%E9%80%9F%E9%A3%9E%E8%89%87%E5%BC%80%E5%A5%96%E7%BB%93%E6%9E%9C%E6%9F%A5%E8%AF%A2%E5%AE%98%E7%BD%91/Gene/Summary?g=ENSLAFG00000003609) |
|  | *Chrysochloris asiatica* | *GBP5* | [101835361](https://www.ncbi.nlm.nih.gov/gene/101835361) | [ENSCASG00000003616](https://www.ensembl.org/Chrysochloris_asiatica/Gene/Summary?g=ENSCASG00000003616) |
|  |  | *GZMB* | [101835362](https://www.ncbi.nlm.nih.gov/gene/101835362) | [ENSCASG00000003615](https://www.ensembl.org/Chrysochloris_asiatica/Gene/Summary?g=ENSCASG00000003615) |
|  |  | *IFNG* | [101835363](https://www.ncbi.nlm.nih.gov/gene/101835363) | [ENSCASG00000003614](https://www.ensembl.org/Chrysochloris_asiatica/Gene/Summary?g=ENSCASG00000003614) |
|  |  | *IRF7* | [101835364](https://www.ncbi.nlm.nih.gov/gene/101835364) | [ENSCASG00000003613](https://www.ensembl.org/Chrysochloris_asiatica/Gene/Summary?g=ENSCASG00000003613) |
|  |  | *KLRD1* | [101835365](https://www.ncbi.nlm.nih.gov/gene/101835365) | [ENSCASG00000003612](https://www.ensembl.org/Chrysochloris_asiatica/Gene/Summary?g=ENSCASG00000003612) |
|  |  | *RTP4* | [101835366](https://www.ncbi.nlm.nih.gov/gene/101835366) | [ENSCASG00000003611](https://www.ensembl.org/Chrysochloris_asiatica/Gene/Summary?g=ENSCASG00000003611) |
|  |  | *TNFSF4* | [101835367](https://www.ncbi.nlm.nih.gov/gene/101835367) | [ENSCASG00000003610](https://www.ensembl.org/Chrysochloris_asiatica/Gene/Summary?g%E6%9E%81%E9%80%9F%E9%A3%9E%E8%89%87%E5%BC%80%E5%A5%96%E7%BB%93%E6%9E%9C%E6%9F%A5%E8%AF%A2%E5%AE%98%E7%BD%91=ENSCASG00000003610) |
|  |  | *TRAT1* | [101835368](https://www.ncbi.nlm.nih.gov/gene/101835368) | [ENSCASG00000003609](https://www.ensembl.org/Chrysochloris_asiatica/Gene/Summary?g=ENSCASG00000003609) |
| Scandentia | *Tupaia belangeri* | *GBP5* | [101936361](https://www.ncbi.nlm.nih.gov/gene/101936361) | [ENSTBEG00000003616](https://www.ensembl.org/Tupaia_belanger%E6%9E%81%E9%80%9F%E9%A3%9E%E8%89%87%E5%BC%80%E5%A5%96%E6%9E%81%E9%80%9F%E9%A3%9E%E8%89%87%E5%BC%80%E5%A5%96%E7%BB%93%E6%9E%9C%E6%9F%A5%E8%AF%A2%E5%AE%98%E7%BD%91/Gene/Summary?g=ENSTBEG00000003616) |
|  |  | *GZMB* | [101936362](https://www.ncbi.nlm.nih.gov/gene/101936362) | [ENSTBEG00000003615](https://www.ensembl.org/Tupaia_belangeri/Gene/Summary?g=ENSTBEG00000003615) |
|  |  | *IFNG* | [101936363](https://www.ncbi.nlm.nih.gov/gene/101936363) | [ENSTBEG00000003614](https://www.ensembl.org/Tupaia_belangeri/Gene/Summary?g=ENSTBEG00000003614) |
|  |  | *IRF7* | [101936364](https://www.ncbi.nlm.nih.gov/gene/101936364) | [ENSTBEG00000003613](https://www.ensembl.org/Tupaia_belangeri/Gene/Summary?g=ENSTBEG00000003613) |
|  |  | *KLRD1* | [101936365](https://www.ncbi.nlm.nih.gov/gene/101936365) | [ENSTBEG00000003612](https://www.ensembl.org/Tupaia_belangeri/Gene/Summary?g=ENSTBEG00000003612) |
|  |  | *RTP4* | [101936366](https://www.ncbi.nlm.nih.gov/gene/101936366) | [ENSTBEG00000003611](https://www.ensembl.org/Tupaia_belangeri/Gene/Summary?g=ENSTBEG00000003611) |
|  |  | *TNFSF4* | [101936367](https://www.ncbi.nlm.nih.gov/gene/101936367) | [ENSTBEG00000003610](https://www.ensembl.org/Tupaia_belangeri/Gene/Summary?g=ENSTBEG00000003610) |
|  |  | *TRAT1* | [101936368](https://www.ncbi.nlm.nih.gov/gene/101936368) | [ENSTBEG00000003609](https://www.ensembl.org/Tupaia_belangeri/Gene/Summary?g=ENST%E6%9E%81%E9%80%9F%E9%A3%9E%E8%89%87%E5%BC%80%E5%A5%96%E7%BB%93%E6%9E%9C%E6%9F%A5%E8%AF%A2%E5%AE%98%E7%BD%910000003609) |
| Dermoptera | *Galeopterus variegatus* | *GBP5* | [102037361](https://www.ncbi.nlm.nih.gov/gene/102037361) |  |
