# Supplementary material for: Structural and evolutionary constraints shape adaptive landscapes of immune-related genes across mammalian phylogeny
Source: PLoS One. 2025 Nov 7;20(11):e0332734. doi: 10.1371/journal.pone.0332734 (PMC12594431; doi:10.1371/journal.pone.0332734)
Supplement: S2 Table — This table provides the NCBI Gene ID and Ensembl Gene ID for the eight immune-related genes analyzed across the 42 mammalian species in this study. These accession numbers serve as unique identifiers for the gene sequences retrieved from the NCBI and Ensembl databases. Blank cells indicate that a standard, curated gene record for that species was not available in the respective database at the time of data collection and an alternative genomic scaffold was used for analysis. (DOCX) [file pone.0332734.s002.docx]

**Supplementary Table S1:** Mapping of Positively Selected Sites to Functional Protein Domains

This table maps the amino acid sites identified under positive selection (Posterior Probability > 0.95 from PAML BEB analysis) to known functional domains and discusses their potential functional implications in the context of host-pathogen co-evolution.

| Gene | Positively Selected Site(s) | Protein Domain (Pfam / InterPro) | Known Domain Function | Potential Functional Implication of Selection |
| --- | --- | --- | --- | --- |
| GBP5 Guanylate Binding Protein 5 | 5, 6, 11, 35, **162**, **215**, 316, 421, 435, 446 | **Guanylate-binding domain** **GTPase domain** | GTP binding and hydrolysis, oligomerization, pathogen membrane targeting. | Sites 162, 215 map to the GTPase domain. Adaptive changes may enhance GTPase activity or alter oligomerization dynamics to improve recognition and disruption of specific pathogen membranes (e.g., from intracellular bacteria). |
| GZMB Granzyme B | 75, 130, 132, 170, 172, **197**, **215**, 223, **237**, 260, 281 | **Trypsin-like serine protease domain (Peptidase S1)** | Cleavage of caspase and other apoptotic substrates in target cells. | Sites 197, 215, 237 are near the catalytic triad. Selection may fine-tune substrate specificity (e.g., for pathogen-encoded caspase inhibitors) or regulate catalytic efficiency to optimize cytotoxic killing of infected cells. |
| IFNG Interferon Gamma | **21**, 54, 99, 116, **120** | **Interferon-gamma cytokine (IFN-g)** | Receptor binding (IFNGR1/IFNGR2), JAK-STAT pathway activation, macrophage activation. | Site 21 is in a flexible loop and site 120 is critical for receptor binding. Mutations could modulate binding affinity to IFNGR, fine-tuning the intensity and duration of the pro-inflammatory immune response to different pathogens. |
| IRF7 Interferon Regulatory Factor 7 | 148, 161, 179, 181, 253, 370, **474** | **DNA-binding domain (IRF)** **IRF association domain (IAD)** | DNA binding (to IFN-stimulated response elements, ISREs), transcription activation, protein-protein interaction. | Site 474 is within a regulatory region. Positive selection could alter transactivation potential or interaction partners (e.g., other IRFs or kinases), modulating the spectrum and strength of antiviral interferon responses. |
| KLRD1 Killer Cell Lectin Like Receptor D1 | 158, 189, 225, 235, 236, 252, **268**, 270, **272**, 274, 276, 289, **290**, **292**, 304 | **C-type lectin domain** | Carbohydrate recognition, binding to MHC-related molecules (e.g., HLA-E in humans), regulates NK cell activity. | Multiple selected sites (e.g., 268, 272, 290, 292) cluster in the ligand-binding interface. This suggests an arms race to recognize rapidly evolving pathogen-derived ligands or altered host MHC molecules presented during infection. |
| RTP4 Receptor Transporter Protein 4 | 25, **53**, 79, 85, 109, 125, 151, 152, 173, **184**, **195**, 196, 199, 208, 235, 249, 261, 282, 393, 403, 406, 409, 470, **479**, 570, 573, 578, 582, 595, 611 | **RTP domain** | Chaperone for odorant and taste receptors, also implicated in trafficking of some interferon-induced GPCRs. | The function in immunity is less defined. Selected sites (e.g., 184, 195, 479) could adapt its chaperone function to assist in the surface expression of a broader or more specific range of immune-related sensory receptors. |
| TNFSF4 TNF Superfamily Member 4 (OX40L) | 19, 64, 158, 223, **235** | **Tumor Necrosis Factor (TNF) domain** | Trimerization, binding to cognate receptor TNFSF4 (OX40), T-cell co-stimulation. | Site 235 is within the TNF homology domain. Selection could modulate the strength of the co-stimulatory signal delivered to T cells, thereby influencing the balance of T-helper cell responses (e.g., Th1/Th2) during chronic infections. |
| TRAT1 T Cell Receptor Associated Transmembrane Adaptor 1 | **12**, 21, 24, 58, **93**, 118, 131 | **Transmembrane adaptor protein** | Scaffold protein in the TCR complex, involved in early signal transduction in T cells. | Sites 12 and 93 are in extracellular/transmembrane regions. Adaptive evolution may adjust the threshold for T-cell activation in response to microbial antigens, preventing autoimmunity or enhancing sensitivity to low-abundance antigens. |
